# Development and Validation of a Food Frequency Questionnaire to Assess Fermented Food Consumption in Adults

**DOI:** 10.1111/jhn.70183

**Published:** 2026-01-14

**Authors:** Aimone Ferri, Elizabeth Schneider, Alice Lucey, Áine Hennessy, Paul D. Cotter, Ramya Balasubramanian, Gerard Clarke, John F. Cryan

**Affiliations:** ^1^ APC Microbiome Ireland University College Cork Cork Ireland; ^2^ Department of Anatomy and Neuroscience University College Cork Cork Ireland; ^3^ School of Food and Nutritional Sciences University College Cork Cork Ireland; ^4^ INFANT Research Centre University College Cork Cork Ireland; ^5^ Food Biosciences Department Teagasc Food Research Centre Cork Ireland; ^6^ Department of Psychiatry and Neurobehavioural Science University College Cork Cork Ireland

**Keywords:** fermentation, food frequency questionnaire, gut health, kefir, kombucha, microbiome, microbiota

## Abstract

**Background:**

Fermented foods can confer benefits to human health and modulate the microbiota–gut–brain axis. Fermented foods are gaining popularity in Western cultures, with increasing calls for their inclusion in national dietary guidelines. As no specific validated measure to capture fermented food intake exists, this study aimed to develop and validate a fermented food intake questionnaire (FFIQ) to assess habitual intake in adults from the United States, Canada, the United Kingdom, Ireland, Australia and New Zealand, aged 18−60 years.

**Methods:**

A 32‐item self‐administered FFIQ, informed by available international food consumption data for adults, was developed and subsequently validated in an online sample of 167 adults using six online 24‐h automated dietary recalls (intake24.com) as the reference method. Correlation and Bland–Altman analyses were used to assess agreement and bias between the FFIQ and the 24‐h dietary recalls.

**Results:**

The most frequently consumed fermented foods were cheeses, yoghurt, kefir and kombucha. Median (Interquartile range) intake of total fermented food was 85.4 (42.3, 143.0) g/day for the FFIQ and 54.9 (20.8, 112.1) g/day for the average of the 24‐h dietary recalls, respectively and showed good agreement for total fermented food consumption (*r* = 0.56, *p* < 0.001) and for most individual fermented foods and food categories. The FFIQ classified 93.4% of participants in the same or adjacent tertile of total fermented food intake. Bland–Altman plots for total intake of fermented food demonstrated good agreement between the FFIQ and the 24 h recalls. The FFIQ also showed good to excellent reliability upon re‐administration for most fermented foods as indicated by the intraclass correlation coefficients.

**Conclusions:**

The FFIQ provides a robust estimate of fermented food consumption among adults from English‐first language countries. This will be a valuable resource with potential applications in clinical and epidemiological research aimed at exploring associations between fermented foods and health outcomes.

## Summary

- A self‐reported 32‐item semi‐quantitative food frequency questionnaire was developed to estimate the consumption of fermented foods within a multi‐country English‐speaking sample and was validated using multiple online 24‐h diet recalls.
- The FFIQ and 24‐h dietary recalls showed good agreement for total fermented food consumption (*r* = 0.56, *p* < 0.001) as well as for most individual fermented foods.
- Foods making the greatest contribution to fermented food intakes include cheeses, yoghurt, kefir and kombucha, while fermented vegetables and fermented meats made lesser contributions.

## Graphical abstract

The fermented food intake questionnaire (FFIQ), developed to assess habitual intake in adults aged 18–60 years, was validated against six 24‐h dietary recalls. The FFIQ showed reasonable validity and repeatability for total fermented food intake as well as for most individual foods and food categories.

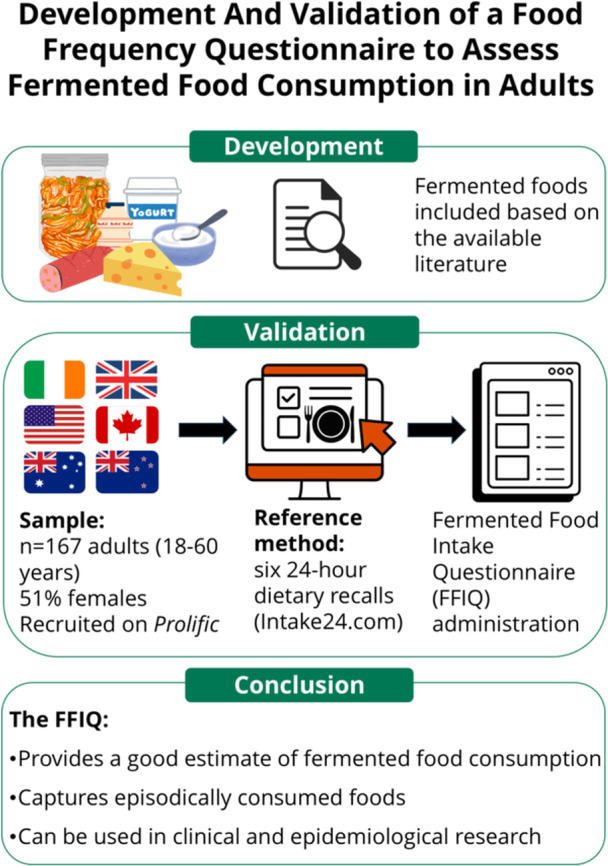

## 1 Introduction

The International Scientific Association for Probiotics and Prebiotics (ISAPP) defines fermented foods as “foods made through desired microbial growth and enzymatic conversions of food components” [1]. The fermentation process mainly relies on yeasts and bacteria. Yeasts such as *Saccharomyces cerevisiae* produce alcohol and carbon dioxide, whereas in the presence of oxygen, they produce acetic acid. Yeasts are widely used in wine and beer brewing as well as in bread production. Among bacteria, the most prevalent are *Acetobacter* and lactic acid bacteria (LAB) from the genera *Leuconostoc, Lactococcus* and *Streptococcus*, along with species formerly classified under the *Lactobacillus* genus [2]. These bacteria are involved in vegetable fermentation, such as in the fermentation of cabbage into sauerkraut, or milk into yoghurt [3]. Bacteria are also involved alongside yeasts in the fermentation of kefir (both water and the milk‐based iterations) and kombucha (a fermented tea beverage). Fermented foods have been consumed for thousands of years across the globe, and fermentation has been used to extend shelf life as well as to modify organoleptic properties. The microorganisms involved in food fermentation are responsible for producing and transforming various compounds, including lactic acid, reduced levels of lactose and other fermentable carbohydrates, increases in certain vitamins (e.g., B12 and K2), amino acids and bioactive peptides (e.g., bacteriocins). Consequently, the fermentation process not only improves shelf‐life, but can also increase the nutritional value of foods [4].

Fermented food consumption has been linked to positive health benefits. For example, evidence from observational studies indicates that the consumption of fermented dairy such as yoghurt is associated with reduced risk of cardiovascular disease [5], type 2 diabetes [6] and depressive symptoms [7]. Dietary interventions with fermented vegetables also report positive health outcomes; for example, kimchi consumption was shown to improve metabolic health and reduce bodyfat in individuals living with overweight or obesity [8]; while sauerkraut consumption improved symptoms of irritable bowel syndrome [9]. Potential mechanisms mediating these beneficial effects can be attributed to the metabolites produced during the fermentation process, but it is also worth noting that fermented foods interact with the gut microbiota [10]. The gut microbiota, that is, the trillions of microorganisms inhabiting the gut, has gained significant research interest, as an altered composition of the gut microbiota has been linked with several pathologies, including metabolic diseases, obesity, cancer, chronic inflammatory diseases, neurodegenerative diseases and mental health conditions [11]. Fermented foods provide a unique opportunity to provide the gut with health‐promoting microorganisms, which can subsequently increase microbiota diversity, reduce inflammation and modulate immune function [12]. Furthermore, the gut microbiota affects brain function through the microbiota–gut–brain axis, by producing bioactive molecules such as short‐chain fatty acids (SCFAs), tryptophan metabolites, and by modulating vagus nerve neurotransmission [13]. The gut–brain axis can be modulated by diet [14], and recent evidence suggests that a gut‐targeted diet including fermented foods has a beneficial effect on perceived stress [15]. Furthermore, recent guidelines on the design and report of clinical trials involving microbiota‐targeted interventions call for a baseline assessment of dietary intakes, including fermented foods, in order to accurately characterise and harmonise the sample where appropriate [16, 17].

However, the lack of available validated tools to reliably assess habitual fermented food intake impedes elucidation of the relationship between fermented foods and health. While food frequency questionnaires (FFQs), or adapted FFQs, are often used in studies [18, 19], these questionnaires are limited by the number or types of fermented food assessed (i.e., yoghurt, cheese and buttermilk) [20]. Other commonly consumed fermented foods in Western cultures (e.g., kefir, kombucha, kimchi and sauerkraut) are frequently not assessed, thus limiting the capacity to capture complete dietary exposure.

Other dietary assessment methods, such as food diaries, while comprehensive in assessing dietary intake, may be burdensome in nature for participants and researchers [21]. A validated fermented food intake questionnaire (FFIQ) that captures episodically consumed foods missed by food diaries or repeated 24 h recalls estimating habitual dietary exposure would be a valuable instrument in clinical research involving fermented foods and microbiota‐targeted interventions.

The aim of this study was to develop and validate a questionnaire to measure FFIQ in adults aged 18–60 years in English‐speaking countries. Specifically, we aimed to develop the FFIQ to:

- Measure the intake of a wide range of fermented foods, which are currently not assessed in extant FFQs.
- Classify individuals based on their total intake of fermented foods.
- Provide a robust tool for use in clinical and epidemiological research to explore the relationship between fermented food intake and health outcomes.

## 2 Methods

### 2.1 Fermented Foods Eligibility Criteria

Fermented foods were defined as foods produced through desired microbial growth and enzymatic conversions of food components (as per the definition established by ISAPP) [1]. Additionally, fermented foods were included if they provided live dietary microorganisms, thus excluding fermented foods with no viable microorganisms present at the time of consumption (i.e., bread, alcoholic products, coffee and cocoa). Consistent with previously published studies [22], pickled items were not included because the majority of pickles are produced without any fermentation process, being preserved instead via acetic acid. Condiments (e.g., soy sauce and miso) were excluded for having very low levels of live microorganisms [22] that are unlikely to yield equivalent magnitudes of benefits. Fermented alcoholic beverages such as wine and beer were excluded from the FFIQ, regardless of containing live microorganisms, due to their recognised adverse effects on health [23]. Fermented foods commonly consumed in each eligible country were selected based on existing literature and the available national food consumption data [24, 25, 26].

### 2.2 Questionnaire Design

The fermented foods selected comprised a wide range of food categories: dairy (including yoghurts, skyr, quark, probiotic shots and kefir), cheeses, fermented non‐dairy alternatives (soy and other plant‐based yoghurt and kefir) vegetables and fruit (sauerkraut, kimchi and olives), soy (tempeh and natto), beverages (kvass, water kefir, tepache and kombucha), fish and seafood (Surströmming and other fermented fish/seafood) and meats (pepperoni, salami, chorizo, summer sausage, prosciutto and Lebanon bologna), and all items were presented with examples of commonly used serving sizes and images. As most fermented foods do not have established serving sizes, portion sizes were based on servings reported by Wastyk et al. [27], in which 6 ounces/175 g constituted one portion of kombucha, yoghurt, kefir and buttermilk and 0.25 cup/40 g for fermented vegetables. Cheese portions were calculated by averaging the national guidelines of the United States, Canada, Australia, New Zealand, the United Kingdom and Ireland (40 g/serving). Portion sizes were listed in ounces, cups and grams to accommodate different international measurements (see Supporting Information File 1 for the FFIQ).

### 2.3 Response Options and Additional Questions

Response options included the following frequencies: ‘Never or less than once per month’; ‘1–3 per month’, ‘Once a week’, ‘2–4 per week’, ‘5–6 per week’, ‘Once a day’, ‘2–3 per day’, ‘4–5 per day’ and ‘6+ per day’. The questionnaire was piloted in a convenience sample (*n* = 10) for refinement prior to validation.

The resulting initial self‐reported FFIQ consisted of 35 different foods and included the option to add a fermented food if not listed in the available options. The questionnaire was administered through *Qualtrics* online in May 2024 to limit disruption to habitual dietary patterns caused by national holidays or periods of religious observation.

Demographics, including age, sex, ethnicity, body mass index (BMI), socioeconomic status, chronic diseases and lifestyle factors (smoking and dietary preferences), were also collected. The SCOFF, a brief validated measure to determine disordered eating symptoms comprising five questions [28], was administered, with scores exceeding 1 indicative of disordered eating (see Supporting Information S2 details of the SCOFF). Feedback regarding questionnaire design and food options was collected from participants at the end of the procedure using closed‐ and open‐ended response formats.

### 2.4 Sample Recruitment

Participants were recruited from Prolific (https://www.prolific.com/), a widely used recruitment platform for online research. It is suggested that a sample size between 100 and 200 would be sufficient for correlation analysis in FFQ validation studies for assessment of dietary intakes [29], and a sample of *n* = 153 would be required to detect the minimum acceptable correlation of *r* = 0.20 [30] with 80% power at *α* = 0.05 (one‐tailed). The final recruited sample after obtaining informed consent was *n* = 197 participants, equally balanced between males and females. Participants were considered eligible to participate if they were aged 18–60 years and citizens and current residents of an English First‐Language country: Australia, Canada, Ireland, New Zealand, the United Kingdom and the United States. Additionally, to ensure scientific rigour, according to the Prolific guidelines, we included two attention checks embedded in the questionnaire. If participants failed both attention checks, their survey would be terminated. Ethical approval was granted by the Clinical Research Ethics Committee of the Cork Teaching Hospitals (CREC) (CREC review reference number ECM 4 (x) 24/10/2023, study number APC182).

### 2.5 Validation Measures

To validate the FFIQ, six multiple‐pass 24‐h recalls were used as the reference measure. Specifically, Intake24 (Intake24.com), an online validated automatic dietary recall containing 2800 foods with images of portion sizes, was used [31]. Two 24‐h dietary recalls are indicated to be sufficient to estimate nutrient intake in a population, and there is rarely a need to exceed five [32]. More recently, a comparison study between two and three 24‐h dietary recalls on consecutive and non‐consecutive days showed that three non‐consecutive days were superior to consecutive days in estimating dietary intake [33]. As our FFIQ aims to capture specific food consumption rather than total macro or micronutrients typically measured in FFQs, combined with an anticipated low frequency of consumption of some fermented foods, we chose to conduct a total of six 24‐h dietary recalls on non‐consecutive days, which included 2 weekend days.

### 2.6 Study Design and Implementation

After providing informed consent, participants completed basic demographic questions. The next day, they were invited to complete their first 24‐h dietary recall on the Intake24 website (https://intake24.com/), where they logged in with their unique anonymised Prolific ID. After the completion of the first dietary recall, they were invited to complete two further 24‐h dietary recalls on non‐consecutive days. To accommodate geographical time differences, participants had 48 h to complete the 24‐h recall before including (or excluding) them from the next dietary recall. The same procedure was completed 10 days later, during which three further non‐consecutive dietary recalls were performed. To account for dietary variability over the weekend, each set of dietary recalls included a weekend day.

**Figure 1:**
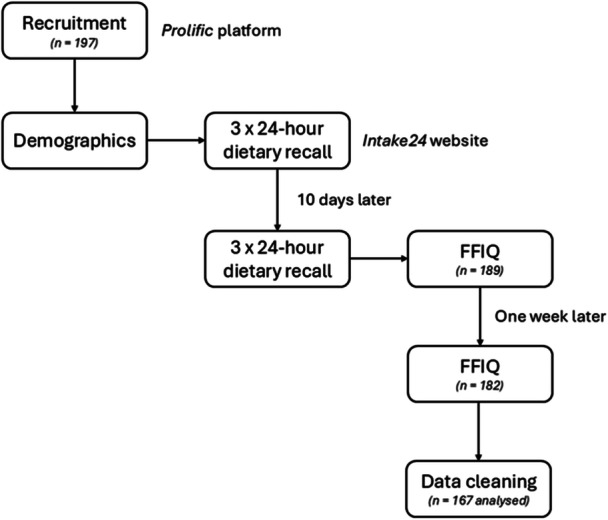
Study flowchart. FFIQ, fermented food intake questionnaire.

Two days after completing the last 24‐h dietary recall, participants were invited to complete the first FFIQ. To assess reliability, 1 week later, participants were asked again to complete the FFIQ (see Figure 1). Participants were compensated according to the Prolific guidelines of a minimum of £6/h.

### 2.7 A Priori Decision on Misreporting of Dietary Intakes and Data Analysis

In accordance with Prolific's policy on attention checks, we planned to exclude from the analysis participants who failed two attention checks embedded within the questionnaire. Participants' data were analysed if they completed the study up to the first FFIQ administration. Participants reporting less than 500 kcal/day or more than 3500 kcal/day in the 24‐h dietary recalls were also excluded [34]. Individual food validation statistics were computed if the level of consumption was available for both the 24 h dietary recalls and the FFIQ.

To obtain more accurate estimates from the FFIQ, particularly for episodically consumed items that may be underreported in 24‐h recalls, foods were aggregated into broader categories based on their similarity in the fermentation process and substrate used, as well as similarity in nutritional composition. Consequently, the following food categories were created: cheese (soft, hard, cream and processed cheeses), fermented dairy (yoghurts, kefir, skyr, quark, Actimel, Yakult and buttermilk), fermented beverages (water kefir, kombucha, ginger beer, beet kvass, probiotic sodas, probiotic juice shots), fermented vegetables and fruit (kimchi, sauerkraut and olives), fermented meats and fish (fermented fish, salami, chorizo, prosciutto crudo, pepperoni, Lebanon bologna and summer sausage) and total intake of fermented foods (sum of all fermented foods). Foods that were not reported in either the 24‐h recalls or the FFIQs were excluded from the validation process.

### 2.8 Data Pre‐Processing

No participant failed their attention checks, and *n* = 189 completed the first FFIQ. After excluding under‐ and over‐reporters and participants with incomplete dietary recall data, the final sample was *n* = 167 participants. Fermented foods not reported by the participants in either the 24‐h recalls or the FFIQ (*n* = 3) were excluded from the validation process and yielded the final 32‐item FFIQ.

Fermented foods used as part of recipes (e.g., kefir smoothie) were extracted and converted into the raw amount of the fermented food used for the recipe according to the recipe breakdown provided by the AUSNUT database tables (https://www.foodstandards.gov.au/science-data/monitoringnutrients/afcd), the same food database that was used by Intake24.com.

Food frequencies from the FFIQ were converted into grams by multiplying the frequency by the standard food portion and divided by 7.

Dailyaverageintake(g)=weeklyfrequencyaverage×serving(g)÷7

for example, if Kimchi (serving = 40 g) was consumed 2–4 times a week:

DailyaverageKimchiintake(g)=3×40g÷7=17.14g

food intake from the 24‐h recalls was averaged by dividing the total amount of food reported by 6 (the total number of dietary recalls) to obtain a daily average.

### 2.9 Validation Statistics

Statistical analyses were conducted using R software version 4.4.1 (34). Histograms were used to visualise the overall shape of the distributions, while QQ plots were used to identify deviations from normality and potential outliers. Validation statistics were conducted on individual food items as well as food categories.

Due to violations of normality assumptions and the skewness of the data, we used non‐parametric statistics. Specifically, to test agreement at the individual level, Spearman's correlation was used alongside Fisher's *z*‐transformation to compute the corresponding confidence intervals. Agreement at the group level was assessed with Wilcoxon's signed‐rank test, and an estimation of the effect size was computed from the *z* value [35]. To assess agreement, direction and magnitude of bias between the two methods, Bland–Altman plots were generated by plotting the pairwise difference against the mean of the two methods. Because the differences were non‐normally distributed, the median of the differences was reported as the measure of bias, and the 95% limits of agreement were defined as the 2.5th and 97.5th percentiles of the differences [36]. To formally assess proportional bias in the Bland–Altman plots, we regressed the differences on the means by using median quantile regression with bootstrapped standard errors as a robust alternative to ordinary least squares regression [37]. As indicated for test‐retest reliability [29], repeatability of the FFIQ was assessed by computing intraclass correlation coefficients (ICCs) using a two‐way mixed‐effects model between the first administration of the FFIQ and the second administration 1 week later. Based upon previous suggestions [38], values less than 0.5, between 0.5 and 0.75, between 0.75 and 0.9, and greater than 0.90 were considered indicative of poor, moderate, good and excellent reliability, respectively. Sensitivity analyses were performed by repeating correlation tests and stratifying the sample by tertiles of age, sex, SCOFF score and country, and the results are reported in Table S1.

## 3 Results

**Figure 2:**
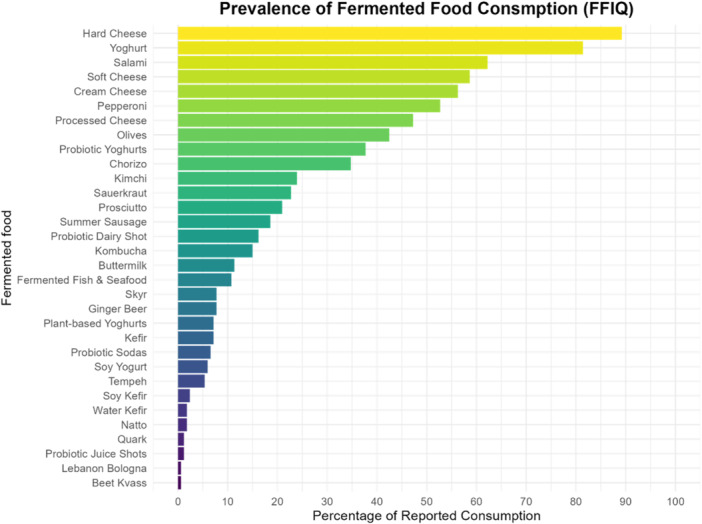
Percentage contributions of individual food items to total fermented food consumption using the FFIQ.

The overall sample mean (SD) age was 38 (11) years, and 51% were females (Table 1 while Table S2 provides additional demographic information). The estimated median (IQR) total intake of fermented food was 85.4 (42.3, 143.0) 71.4 g/day for the FFIQ and 54.9 (20.8, 112.1) g/day for the average of the 24‐h recalls, respectively. According to the FFIQ, the most consumed fermented foods were hard cheese (89.2%), yoghurt (81.4%), salami (62.3%), soft cheese (58.7%) and cream cheese (56.3%) (Figure 2). The level of consumption of some fermented foods was very low, with multiple foods displaying a median intake of zero (Table 2).

**Table 1:** Final sample demographics.

Characteristic	*N* = 167
Age (years)^a^	38 (11)
Country of residence	
Australia	36 (22%)
Canada	43 (26%)
Ireland	22 (13%)
New Zealand	19 (11%)
United Kingdom	20 (12%)
United States	27 (16%)
Sex	
F	85 (51%)
M	82 (49%)
Ethnicity	
White	134 (80%)
Asian	21 (13%)
Black	4 (2.4%)
Mixed	7 (4.2%)
Other	1 (0.6%)

^a^ Mean (SD); *n* (%).

**Table 2:** Summary statistics of fermented food intake reported in both the FFIQ and multiple 24‐h recalls (g/day) in a sample of adults (*n* = 167). Aggregated variables for the FFIQ include foods not reported in the multiple 24‐h recalls.

	Mean (SD) FFIQ intake	Mean (SD) 24‐h recall intake	Median (IQR) FFIQ intake	Median (IQR) 24‐h recall intake
Fermented food				
Yoghurt	48.5 (79.6)	45.2 (82.3)	17.9 (8.9, 53.6)	10.8 (0, 59.6)
Hard cheese	19.7 (24.6)	7.5 (10.1)	17.1 (2.9, 24.3)	3.5 (0, 11.4)
Soft cheese	4.4 (9.5)	11.5 (15.9)	2.9 (0, 5.7)	3.4 (0, 20.6)
Cream cheese	2.5 (4)	1.7 (9.4)	2.1 (0, 2.1)	0 (0, 0)
Chorizo	1.1 (2.7)	0.2 (1.6)	0 (0, 2.1)	0 (0, 0)
Salami	2.7 (4.4)	1.2 (6.2)	2.1 (0, 2.1)	0 (0, 0)
Pepperoni	2.1 (3.6)	4.1 (8.8)	2.1 (0, 2.1)	0 (0, 0)
Kefir	6.4 (37.8)	4.5 (28.2)	0 (0, 0)	0 (0, 0)
Kimchi	2 (8.8)	0.9 (5.9)	0 (0, 0)	0 (0, 0)
Kombucha	5.1 (20.2)	9.2 (55.6)	0 (0, 0)	0 (0, 0)
Water kefir	1 (8.2)	0.3 (3.3)	0 (0, 0)	0 (0, 0)
Sauerkraut	1.1 (3.3)	0.8 (3.9)	0 (0, 0)	0 (0, 0)
Probiotic dairy shot	4 (22.6)	0.5 (4.5)	0 (0, 0)	0 (0, 0)
Plant‐based yoghurt	2.5 (13)	1.1 (13.2)	0 (0, 0)	0 (0, 0)
Olives	1.4 (3.5)	0.3 (2.3)	0 (0, 1.1)	0 (0, 0)
Ginger beer	4.3 (34.8)	0.7 (6.3)	0 (0, 0)	0 (0, 0)
Fermented food categories				
Cheeses	27.4 (32.3)	20.8 (19.6)	19.3 (7.6, 37.6)	17.2 (5.2, 29.3)
Fermented beverages	11.9 (44.2)	10.2 (59.3)	0 (0, 0)	0 (0, 0)
Fermented dairy	81.2 (145.9)	50.2 (86.4)	53.6 (8.9, 82.0)	12.3 (0, 73)
Fermented vegetables	4.6 (11.4)	2.0 (7.9)	1.1 (0, 5)	0 (0, 0)
Fermented meats and fish	5.5 (12.6)	5.5 (10.7)	4.3 (2.1, 8.6)	0 (0, 6.9)
Total fermented food	141.4 (214.5)	89.8 (111)	85.4 (42.3, 143.0)	54.9 (20.8, 112.1)

Abbreviations: IQR = interquartile range, SD = standard deviation.

### 3.1 Total Fermented Food Intake

**Figure 3:**
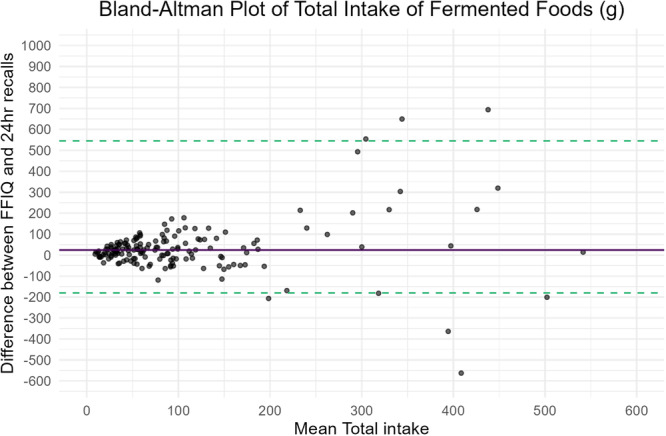
Bland–Altman plot of total fermented food intake (g/day). The green dashed lines represent the upper (97.5th percentile) and the lower (2.5th percentile) limit of agreement. The solid blue line represents the bias (median difference of intake).

Total fermented food intake from the FFIQ and the 24‐h recalls were strongly correlated (*r* = 0.56, *p* < 0.001). Differences at the group level were statistically significant, with a moderate effect size (*W* = 10,085, *r* = 0.38, *p* < 0.001), indicating that the FFIQ slightly overestimates intakes. According to the Bland–Altman plot, a positive bias for the FFIQ was detected with a median (IQR) difference of 24.20 g (−11.42, 62.63). All but 10 participants fell within the limits of agreement, with no proportional bias (slope = 0.38, 95% CI = −0.08, 0.84; *p* = 0.11) but with wide limits of agreement (Figure 3).

The FFIQ classified 93.4% of participants in the same or adjacent tertile of total fermented food intake. Results of the validation statistics for total fermented food intake, food categories and individual foods are presented in Table 3, and the additional Bland–Altman plots for the food categories are provided as Figures S2–S6. Statistics were repeated with the exclusion of an outlier reporting > 2000 g of total fermented food/day, but no appreciable differences in the validation statistics were observed (data not shown). Sensitivity analyses showed good agreement overall between the FFIQ and the 24‐h recalls when stratified by sex, age and risk of eating disorders according to the SCOFF screening tool (Table S2).

**Table 3:** Summary of the validation statistics between the FFIQ and the 24‐h recalls.

	Spearman's correlation statistics			Wilcoxon's signed‐rank test	
Variable	Spearman's *ρ*	95% CI	*p*	Test statistic (*W*)	*p*
Cheeses	0.19	0.04, 0.33	0.013	7775.0	0.05
Hard cheese	0.50	0.37, 0.6	< 0.001	10,136.5	< 0.001
Soft cheese	0.09	−0.07, 0.24	0.26	2213.0	< 0.001
Cream cheese	0.31	0.16, 0.44	< 0.001	3730.0	< 0.001
Fermented dairy	0.67	0.58, 0.75	< 0.001	8153.0	< 0.001
Yoghurt	0.68	0.59, 0.75	< 0.001	5743.0	0.0647
Milk kefir	0.65	0.56, 0.73	< 0.001	62.0	0.0758
Probiotic dairy shot	0.35	0.21, 0.47	< 0.001	324.0	< 0.001
Fermented beverages	0.41	0.28, 0.53	< 0.001	603.0	0.024
Kombucha	0.51	0.39, 0.62	< 0.001	195.0	0.621
Water kefir	0.58	0.47, 0.67	< 0.001	6.0	0.181
Ginger beer	0.17	0.02, 0.31	0.028	84.0	0.0468
Fermented vegetables	0.38	0.24, 0.5	< 0.001	4002.0	< 0.001
Sauerkraut	0.40	0.26, 0.52	< 0.001	549.0	0.0223
Kimchi	0.45	0.32, 0.57	< 0.001	634.0	< 0.001
Olives	0.15	0, 0.29	0.058	2313.0	< 0.001
Fermented meats	0.43	0.3, 0.54	< 0.001	6139.0	< 0.001
Chorizo	0.23	0.08, 0.37	0.0031	1606.0	< 0.001
Salami	0.26	0.11, 0.39	< 0.001	4791.0	< 0.001
Pepperoni	0.42	0.28, 0.54	< 0.001	1897.0	0.201
Plant‐based yoghurt	0.33	0.19, 0.46	< 0.001	191.0	< 0.001
Total fermented food	0.56	0.44, 0.65	< 0.001	10,085.0	< 0.001

### 3.2 Fermented Food Categories

No observed proportional biases were present for any of the food categories, as assessed by regressing the median difference in estimates on the median of the two methods (all *p* > 0.05). Additionally, all but 10 participants fell within the limits of agreement for each food category.

### 3.3 Fermented Fruit and Vegetables

A moderate correlation was observed between the FFIQ and the 24‐h recalls (*r* = 0.38, *p* < 0.001). Statistically significant differences were observed at group level (*W* = 4188, *r* = −0.57, *p* < 0.001) with a large effect size. According to the Bland–Altman plot a positive bias for the FFIQ was detected with a median (IQR) difference of 1.1 (0, 2.6) g.

### 3.4 Fermented Dairy Products

A strong, significant correlation was observed between the FFIQ and 24‐h recalls (*r* = 0.67, *p* < 0.001). A statistically significant difference between the FFIQ and the 24‐h recall was detected (*W* = 8153, *r* = 0.43, *p* < 0.001) with a moderate effect size. The Bland–Altman plot showed a positive bias for the FFIQ with a median (IQR) difference of 8.9 (0, 31) g.

### 3.5 Beverages

A moderate, significant correlation was observed for the beverages category (*r* = 0.41, *p* < 0.001). A statistically significant difference between the FFIQ and the 24‐h recall was detected (*W* = 603, *r* = 0.35, *p* = 0.024) with a moderate effect size. The Bland–Altman plot showed no bias between the FFIQ and the 24‐h recall.

### 3.6 Meats

A moderate, significant correlation was observed between the FFIQ and the 24‐h recalls (*r* = 0.43, *p* < 0.001). A significant difference in the intakes measured by the two methods was detected (*W* = 6139, *r* = 0.24, *p* = 0.001) but with a small effect size. The Bland–Altman plot showed a positive bias for the FFIQ with a median (IQR) difference of 2.14 (0, 6.42) g.

### 3.7 Cheeses

A small, significant correlation was observed between the FFIQ and the 24‐h recalls (*r* = 0.19, *p* = 0.013). A statistically significant difference between the FFIQ and the 24‐h recall was detected (*W* = 7775, *r* = −0.154, *p* = 0.049) with a small effect size. The Bland*–*Altman plot showed a positive bias for the FFIQ with a median (IQR) difference of 4.43 (−10.67, 17.23) g.

### 3.8 Reliability Analysis

Reliability of the questionnaire through repeat administration showed satisfactory overall reliability, with most items displaying good to excellent agreement (ICC > 0.75). Probiotic sodas, probiotic juice shot, soy kefir, beet kvass, Lebanon bologna and processed cheese demonstrated poor reliability (ICC < 0.5), indicating that their consumption was inconsistently reported between the two time points. The summary of the intraclass correlation coefficients is presented in Table 4.

**Table 4:** Summary of the FFIQ reliability and reproducibility.

Variable	Intraclass correlation statistics, two‐way mixed‐effects model		
	ICC	95% CI lower	95% CI upper
Kefir	0.95	0.93	0.96
Water kefir	0.94	0.92	0.96
Kombucha	0.94	0.92	0.95
Kimchi	0.93	0.9	0.95
Sauerkraut	0.91	0.88	0.94
Olives	0.91	0.88	0.94
Tempeh	0.88	0.84	0.91
Yoghurt	0.87	0.83	0.91
Salami	0.87	0.82	0.9
Probiotic dairy shot	0.86	0.81	0.90
Pepperoni	0.85	0.79	0.89
Cream cheese	0.84	0.79	0.88
Natto	0.84	0.78	0.88
Prosciutto	0.83	0.77	0.88
Probiotic yoghurt	0.82	0.75	0.86
Ginger beer	0.82	0.76	0.87
Chorizo	0.82	0.76	0.87
Buttermilk	0.79	0.73	0.85
Hard cheese	0.79	0.73	0.85
Soft cheese	0.75	0.66	0.82
Summer sausage	0.69	0.58	0.77
Quark	0.64	0.51	0.73
Fermented fish	0.57	0.42	0.69
Skyr	0.55	0.39	0.67
Plant‐based yoghurt	0.54	0.38	0.66
Soy yoghurt	0.52	0.35	0.65
Probiotic sodas	0.37	0.15	0.54
Probiotic juice shot	0.32	0.05	0.49
Soy kefir	0.26	0.00	0.46
Beet kvass	0.00	−0.36	0.26
Lebanon bologna	0.00	−0.36	0.26
Processed cheese	−0.04	−0.41	0.23

## 4 Discussion

This study describes the creation and validation of an FFIQ designed to capture dietary exposure for a variety of fermented foods. Novel aspects of the FFIQ are the use of an online recruitment platform (*Prolific*), which enabled the recruitment of a diverse sample across six English‐speaking countries, and the use of a comprehensive online 24‐h dietary recall tool (*Intake24*) as the reference measure.

Previous studies have attempted to develop or validate a questionnaire to assess fermented food intake. However, these efforts have often yielded a limited number of foods [20] or lack a formal validation [39], highlighting the need for a reliable tool to estimate the consumption of a variety of fermented foods in the population. Unlike prior FFQs, the FFIQ has the additional capacity to estimate intakes of a wider range of fermented foods, including vegetables, kefir and kombucha, which have not been captured previously. Capturing intakes of fermented vegetables and fermented plant‐based dairy alternatives is especially relevant given the increasing prevalence of vegan and dairy‐free diets, their distinct nutritional profile and the different microbial communities involved in the fermentation of vegetables [40]. In addition to capturing the most consumed fermented foods (e.g., cheese and fermented meats), the FFIQ had the capability to capture low‐frequency consumption of less commonly consumed items, such as non‐dairy fermented beverages and fermented vegetables. As the FFIQ was developed and validated in a diverse sample of English‐speaking adults from the United Kingdom, Ireland, the United States, Canada, Australia and New Zealand, with an equal distribution of males and females, and included culturally relevant examples alongside pictures of serving sizes for each fermented food, these design considerations support its applicability across multiple English‐speaking populations.

### 4.1 Methodological Considerations

Although the FFIQ tended to overestimate fermented food consumption on average, this difference appears to be modest in practical terms. Most of the food items had satisfactory correlation coefficients between the FFIQ and the 24‐h recalls, for example, yoghurt, kefir, kombucha as well as the fermented dairy category and ‘total intake of fermented food’ demonstrated the strongest correlations (*r* > 0.5). For yoghurt and fermented dairy, these strong correlations were also reported by Li et al. [20], who developed an FFQ to assess fermented food intake in the Dutch population, thus indicating consistent validity of these items. The Bland–Altman plots report an acceptable level of agreement between the two methods, with no proportional bias overall. A positive fixed bias was observed in favour of the FFIQ compared to the 24‐h recalls, but this bias appears small in practical terms (range: 2.14–10.49 g/day) when compared to the average intake of fermented foods, and it is similar to what was previously reported. Heteroscedasticity in the data points was detected, as shown by an increased scattering of the observations when the mean intake increases, suggesting lower accuracy in estimating very high intakes of fermented foods. This may be due to some participants reporting high intakes of fermented foods but with a low weekly frequency, which resulted in the food items captured only by either the FFIQ or 24‐h recall; or it may be a result of random errors from the participants in reporting their food intake. Finally, cross‐classification into tertiles of total fermented food intake yielded satisfactory results, with only 7.2% of participants misclassified into opposite tertiles of intake.

While most food items showed acceptable validation statistics, some food items showed weaker findings. For example, soft cheese had a surprisingly low correlation coefficient that may be related to the order in which the food items were presented (soft cheese first, then cream cheese): participants may have confused soft cheese with cream cheese despite our questionnaire providing examples of such types of cheese. Another possibility is a recall bias, as most of the soft cheese consumed was consumed through pizza (made with mozzarella). Unsatisfactory correlations for ginger beer may be due to misreporting as consumption of ginger ale instead of ginger beer, as the two terms are often colloquially used interchangeably despite different beverages: ginger beer is a fermented beverage, whereas ginger ale is a flavoured carbonated beverage that does not require a fermentation process. Finally, low ICC observed in the reliability analysis for probiotic sodas, probiotic juice shot, soy kefir, beet kvass, Lebanon bologna and processed cheese, suggest that their intake may not be reliably measured. This may reflect their relatively infrequent or irregular consumption, which may have affected participants' ability to recall the intake of these foods upon the second administration, and thus, their removal from the final FFIQ is warranted.

### 4.2 Strengths and Limitations

The 32‐item FFIQ has several strengths. Firstly, the entire data collection process was completed online, which allowed us to reach geographically distant countries, but with a shared language and similar food habits. The recruitment of a balanced sample between males and females is another strength as it ensures the FFIQ retains its validity for both sexes. Moreover, the recruitment of a large sample ensured the study was sufficiently powered to capture intra‐ and inter‐individual variability in food intake. However, due to the low level of consumption of some fermented foods, it was not feasible to construct Bland‐Altman plots for all the food items. To address the issue, Bland–Altman plots were constructed for aggregated variables, which enabled a more comprehensive estimate of consumption at the food group level. Another possible limitation is that episodically consumed fermented foods may not have been reliably captured by the total of six dietary recalls, despite the fact that it is suggested that, in most instances, two repeated dietary recalls are sufficient to describe food intake [32]. This discrepancy is expected because of investigating specific food items rather than total macronutrients or micronutrients.

Several sensitivity analyses were conducted to strengthen the results. Stratification by sex, age, country of residence and SCOFF score did not substantially alter the overall results. Specifically, correlations among females remained consistent, which is notable given that misreporting of food intake has been reported more commonly in females than in males [41].

Finally, while the sample size of *n* = 167 obtained for the validation exceeds the minimum sample size of *n* = 153 (described in Section Methods, 2), thus providing reasonable power for the main validation analyses, smaller associations or subgroup‐specific effects may not be captured. Future research should aim to complement the validation of the FFIQ with objective biomarkers of fermented food intake, although such markers are currently not available [42]. Furthermore, the present study focused on a relatively homogeneous sample, and a formal external validation analysis was not conducted. However, the validity and consistency of the FFIQ were addressed through sensitivity analyses limited to age, sex, country of residence,\ and SCOFF score. Replication in more diverse populations and settings would be valuable to confirm generalisability across different contexts.

In summary, fermented food consumption has gained significant interest in recent years. Current knowledge of habitual intakes is still limited, impeded by the low availability of accurate standardised dietary assessment tools. The development of a validated FFIQ to assess consumption patterns is necessary to accurately capture dietary exposure to these foods, which also considers the establishment of fermented food serving sizes [43]. We have shown that the FFIQ is a useful tool for assessing commonly consumed fermented foods, and it effectively classifies low and high levels of intake. Currently available and widely employed dietary pattern indices, such as the Healthy Eating Index, do not adequately capture fermented food intake; therefore, the FFIQ could be employed to evaluate fermented food consumption in relation to health outcomes [44]. Another area of application is classifying individuals based on their intake of fermented food groups for microbiota‐based and dietary intervention studies to appropriately characterise and stratify the sample, as habitual intake of fermented foods can affect the gut microbiome, dietary responses, and consequently the interpretation of the intervention effects. Similarly, observational studies investigating the role of diet and fermented foods in the microbiota–gut–brain axis, and more generally nutritional epidemiology, will benefit from implementing the FFIQ. It provides a detailed measure of fermented foods intake that is otherwise difficult to capture through a limited number of 24‐h dietary recalls, and it can be used alongside to adjust for day‐to‐day variability in episodically consumed foods [45]. This, in turn, could strengthen the evidence base linking fermented foods and human health, to inform future dietary recommendations.

## 5 Conclusion

In conclusion, this FFIQ represents a valuable dietary tool to investigate fermented food consumption in an adult population of English‐speaking countries with wide applications in research. This FFIQ demonstrates good validity and reproducibility and can be readily employed to provide a robust estimate of fermented foods consumption and to identify key foods contributing to total fermented food intakes in the diet. This FFIQ will have a wide range of applications in clinical and epidemiological research.

## Author Contributions

**Aimone Ferri:** conceptualisation, methodology, formal analysis, investigation, project administration, visualisation, writing – original draft. **Elizabeth Schneider:** conceptualisation, methodology. **John F. Cryan:** conceptualisation, methodology, funding acquisition. **Alice Lucey:** Methodology. **Áine Hennessy:** Methodology. **Paul D. Cotter:** Methodology. **Ramya Balasubramanian:** Methodology. **Gerard Clarke:** Methodology. All authors reviewed, revised and approved the final version of the manuscript.

## Ethics Statement

Ethical approval was granted by the Clinical Research Ethics Committee of the Cork Teaching Hospitals (CREC) (CREC review reference number ECM 4 (x) 24/10/2023, study number APC182).

## Conflicts of Interest

E.S. has received honorarium from Janssen Sciences Ireland UC. E.S. also received an honorarium from MyNutriWeb for an event that was sponsored by Yakult. The laboratory directed by P.D.C. has received research funding from FrieslandCampina, PrecisionBiotics Group, PepsiCo and Danone. P.D.C. has also received support from PepsiCo, Arla, Danone, Yakult, AG1 and H&H to attend and speak at scientific conferences and other events. In addition, P.D.C. is a co‐founder and serves as Head of Microbiology of SeqBiome. G.C. has received honoraria from Janssen, Probi, Apsen and Ingelheim Boehringer as an invited speaker; is in receipt of research funding from Pharmavite, Fonterra, Reckitt, Nestle and Tate and Lyle; and has been paid for consultancy work by Yakult, Zentiva, Bayer Healthcare and Heel Pharmaceuticals. J.F.C. has been an invited speaker at conferences organised by Bromotech, Yakult, Nutricia and Nestle and has received research funding from Kerry Foods, DuPont/IFF and Nestle. The other authors declare no conflicts of interest.

## Supporting information

Supporting file 1 fermented food intake questionnaire.docx.

Supplementary Figure 1. Percentage contributions of selected fermented food groups to total fermented food consumption reported in the FFIQ by country. Supplementary Figure 2. Fermented vegetables. Supplementary Figure 3. Fermented dairy intake. Supplementary Figure 4. Fermented beverages intake. Supplementary Figure 5. Fermented meats intake. Supplementary Figure 6. Cheese (total) intake.
