# Supplementary material for: Development and Validation of a Food Frequency Questionnaire to Assess Fermented Food Consumption in Adults
Source: J Hum Nutr Diet. 2026 Jan 14;39(1):e70183. doi: 10.1111/jhn.70183 (PMC12801178; doi:10.1111/jhn.70183)
Supplement: Supplementary file 1 — Supporting file 1 fermented food intake questionnaire.docx. [file JHN-39-0-s002.docx]

Fermented Food Intake Questionnaire

**Fermented Dairy - Yogurt** *Remember to include yogurt when you have it****on its own*** *and when you use it****as part of recipes*** *(e.g., oats and yogurt, marinating meats with yogurt, salad dressings, Lassi etc.).* **How many times did you consume one serving of Yogurt in the past 30 days?**


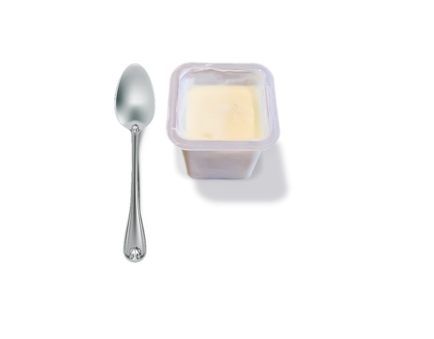


|  | Never or less than once per month | 1-3 per month | Once a week | 2-4 per week | 5-6 per week | Once a day | 2-3 per day | 4-5 per day | 6+ per day | I don’t know what this item is |
| --- | --- | --- | --- | --- | --- | --- | --- | --- | --- | --- |
| ⊗Yogurt and Greek yogurt (any type, 1 pot or 125g or 4.4oz) |  |  |  |  |  |  |  |  |  |  |

**Fermented Dairy - Probiotic Yogurt (e.g., Activia brand)** *Remember to include yogurt when you have it****on its own*** *and when you use it****as part of recipes*** *(e.g., oats and yogurt, marinating meats with yogurt, salad dressings, Lassi etc.).* **How many times did you consume one serving of probiotic Yogurt in the past 30 days?**


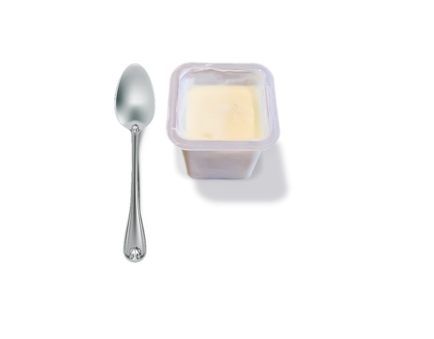


|  | Never or less than once per month | 1-3 per month | Once a week | 2-4 per week | 5-6 per week | Once a day | 2-3 per day | 4-5 per day | 6+ per day | I don’t know what this item is |
| --- | --- | --- | --- | --- | --- | --- | --- | --- | --- | --- |
| ⊗Probiotic yogurt (any type, 1 pot or 125g or 4.4oz) |  |  |  |  |  |  |  |  |  |  |

**Fermented Dairy - Skyr** Remember to include Skyr (Icelandic yogurt) when you have it on its own and when you use it as part of recipes (e.g., oats and yogurt, marinating meats with yogurt, salad dressings etc.). **How many times did you consume one serving of Skyr in the past 30 days?**


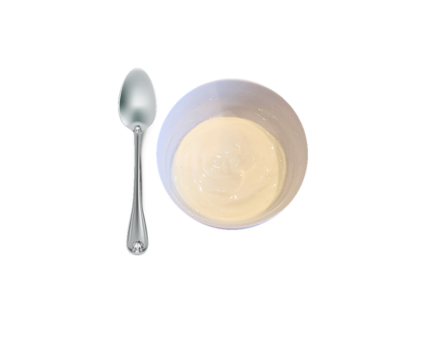


|  | Never or less than once per month | 1-3 per month | Once a week | 2-4 per week | 5-6 per week | Once a day | 2-3 per day | 4-5 per day | 6+ per day | I don’t know what this item is |
| --- | --- | --- | --- | --- | --- | --- | --- | --- | --- | --- |
| ⊗Skyr (1 pot or 150g or 5.3oz) |  |  |  |  |  |  |  |  |  |  |

**Fermented Dairy - Kefir** Remember to include milk kefir when you have it on its own and when you use it as part of recipes (e.g., oats and yogurt, marinating meats with yogurt, salad dressings etc.). **How many times did you consume one serving of milk kefir in the past 30 days?**


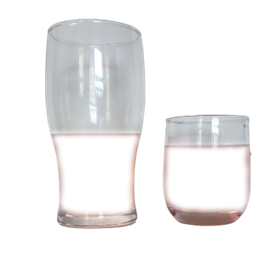


|  | Never or less than once per month | 1-3 per month | Once a week | 2-4 per week | 5-6 per week | Once a day | 2-3 per day | 4-5 per day | 6+ per day | I don’t know what this item is |
| --- | --- | --- | --- | --- | --- | --- | --- | --- | --- | --- |
| ⊗Milk kefir (1 glass or 3/4 of a cup or 175ml or 6 oz) |  |  |  |  |  |  |  |  |  |  |

**Fermented Dairy Shots - (e.g., Yakult or Actimel brands)** Remember to include Yakult when you have it on its own and when you use it as part of recipes (e.g., oats and yogurt, marinating meats with yogurt, salad dressings etc.). **How many times did you consume one serving of Yakult in the past 30 days?**


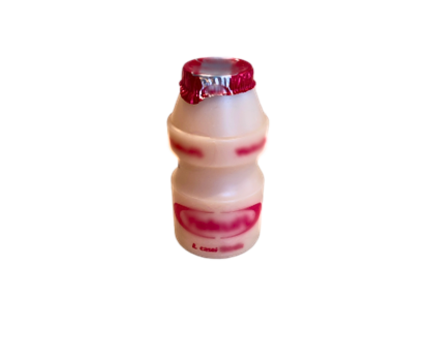


|  | Never or less than once per month | 1-3 per month | Once a week | 2-4 per week | 5-6 per week | Once a day | 2-3 per day | 4-5 per day | 6+ per day | I don’t know what this item is |
| --- | --- | --- | --- | --- | --- | --- | --- | --- | --- | --- |
| ⊗Yakult (1 bottle) |  |  |  |  |  |  |  |  |  |  |

**Fermented Dairy - Buttermilk** Remember to include buttermilk when you have it on its own and when you use it as part of recipes (e.g., oats and yogurt, marinating meats with yogurt, salad dressings etc.). **How many times did you consume one serving of Buttermilk in the past 30 days?**


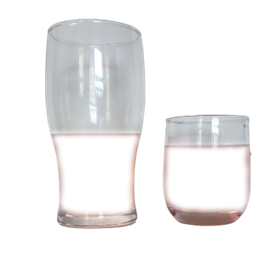


|  | Never or less than once per month | 1-3 per month | Once a week | 2-4 per week | 5-6 per week | Once a day | 2-3 per day | 4-5 per day | 6+ per day | I don’t know what this item is |
| --- | --- | --- | --- | --- | --- | --- | --- | --- | --- | --- |
| ⊗Buttermilk (1 glass or 3/4 of a cup or 175ml) |  |  |  |  |  |  |  |  |  |  |

**Fermented Dairy - Quark** Remember to include Quark cheese/Kvarg when you have it on its own and when you use it as part of recipes (e.g., oats and yogurt, marinating meats with yogurt, salad dressings etc.). **How many times did you consume one serving of quark/kvarg in the past 30 days?**


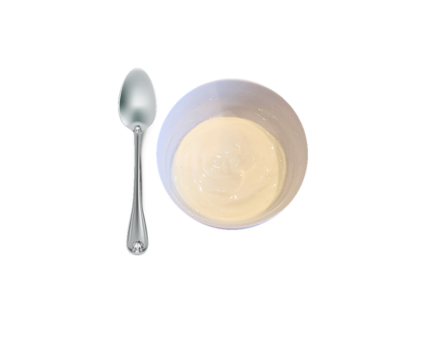


|  | Never or less than once per month | 1-3 per month | Once a week | 2-4 per week | 5-6 per week | Once a day | 2-3 per day | 4-5 per day | 6+ per day | I don’t know what this item is |
| --- | --- | --- | --- | --- | --- | --- | --- | --- | --- | --- |
| ⊗Quark/Kvarg (1 pot or 150g or 5.3 oz) |  |  |  |  |  |  |  |  |  |  |

**Fermented dairy alternatives - soy yogurt** Remember to include soy yogurt when you have it **on its own** and when you use it **as part of recipes** (e.g., oats and yogurt). **How many times did you consume one serving of soy yogurt in the past 30 days?**


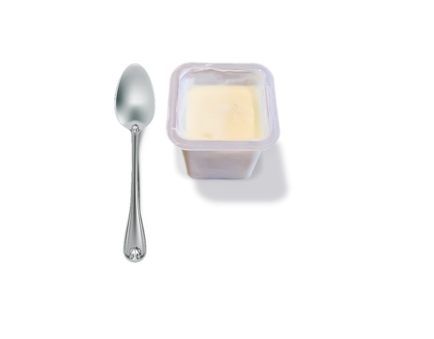


|  | Never or less than once per month | 1-3 per month | Once a week | 2-4 per week | 5-6 per week | Once a day | 2-3 per day | 4-5 per day | 6+ per day | I don’t know what this item is |
| --- | --- | --- | --- | --- | --- | --- | --- | --- | --- | --- |
| ⊗Soy yogurt (1 pot or 125g or 4.4 oz) |  |  |  |  |  |  |  |  |  |  |

**Fermented dairy alternatives - other non-dairy yogurt** Remember to include other non-dairy yogurt when you have them **on their own** and when you use them **as part of recipes** (e.g., oats and yogurt). **How many times did you consume one serving of other non-dairy yogurt in the past 30 days?**


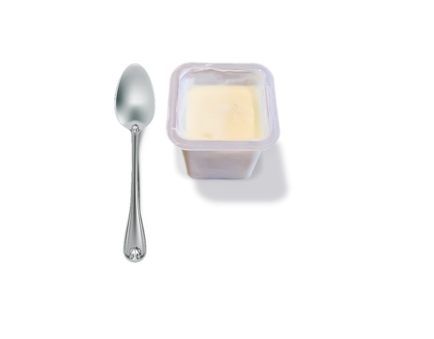


|  | Never or less than once per month | 1-3 per month | Once a week | 2-4 per week | 5-6 per week | Once a day | 2-3 per day | 4-5 per day | 6+ per day | I don’t know what this item is |
| --- | --- | --- | --- | --- | --- | --- | --- | --- | --- | --- |
| ⊗Other non-dairy yogurt (e.g., almond, coconut, cashew etc., 1 pot or 125g or 4.4 oz) |  |  |  |  |  |  |  |  |  |  |

**Fermented dairy alternatives - Soy kefir** Remember to include Soy kefir when you have it **on its own** and when you use it **as part of recipes** (e.g., oats and yogurt). **How many times did you consume one serving of Soy kefir in the past 30 days?**


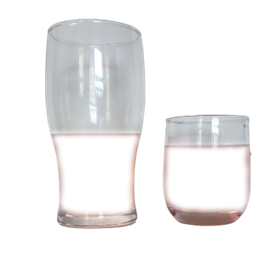


|  | Never or less than once per month | 1-3 per month | Once a week | 2-4 per week | 5-6 per week | Once a day | 2-3 per day | 4-5 per day | 6+ per day | I don’t know what this item is |
| --- | --- | --- | --- | --- | --- | --- | --- | --- | --- | --- |
| ⊗Soy kefir (1 glass or 3/4 of a cup or 175ml) |  |  |  |  |  |  |  |  |  |  |

**Hard or semi hard cheese** Remember to include hard or semi-hard cheeses when you have them **on their own** and when you use them **as part of recipes** (e.g., pizza, toast, sandwiches, dips etc.) **How many times did you consume one serving of hard or semi-hard cheese in the past 30 days?**


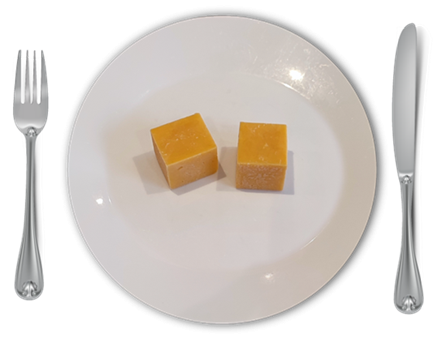


|  | Never or less than once per month | 1-3 per month | Once a week | 2-4 per week | 5-6 per week | Once a day | 2-3 per day | 4-5 per day | 6+ per day | I don’t know what this item is |
| --- | --- | --- | --- | --- | --- | --- | --- | --- | --- | --- |
| Hard or semi-hard cheese (40g, two AA batteries, or 2 slices). Examples: Cheddar, Gouda, Parmesan, Pecorino, Provolone, Edam, Muenster, Asiago, Colby, Monterey Jack, Feta, Halloumi. |  |  |  |  |  |  |  |  |  |  |

**Processed cheese slices/ American cheese slices** Remember to include processed cheese slices/ American cheese slices (e.g., Kraft brand) when you have them **on their own** and when you use them **as part of recipes** (e.g., pizza, toast, sandwiches, cheeseburger, dips etc.) **How many times did you consume one serving of Processed cheese slices/ American cheese slices in the past 30 days?**


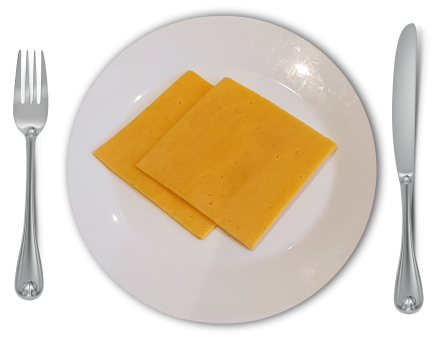


|  | Never or less than once per month | 1-3 per month | Once a week | 2-4 per week | 5-6 per week | Once a day | 2-3 per day | 4-5 per day | 6+ per day | I don’t know what this item is |
| --- | --- | --- | --- | --- | --- | --- | --- | --- | --- | --- |
| Processed cheese slices/American cheese 1 slice (22g) |  |  |  |  |  |  |  |  |  |  |

**Soft cheese** Remember to include soft cheeses when you have them **on their own** and when you use them **as part of recipes** (e.g., pizza, toast, sandwiches, dips etc.) **How many times did you consume one serving of soft cheese in the past 30 days?**


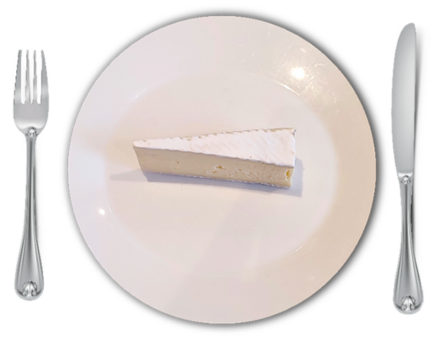


|  | Never or less than once per month | 1-3 per month | Once a week | 2-4 per week | 5-6 per week | Once a day | 2-3 per day | 4-5 per day | 6+ per day | I don’t know what this item is |
| --- | --- | --- | --- | --- | --- | --- | --- | --- | --- | --- |
| Soft cheese (40g or two AA batteries). Examples: Brie, Mozzarella, Cottage cheese, Goats’ cheese, Blue/Stilton/Roquefort, Camembert. |  |  |  |  |  |  |  |  |  |  |

**Cream cheese and sour cream** Remember to include cream cheeses or soured creams when you have them on their own and when you use them as part of recipes (e.g., pizza, toast, sandwiches, dips etc.) **How many times did you consume one serving of cream cheeses/soured creams in the past 30 days?**


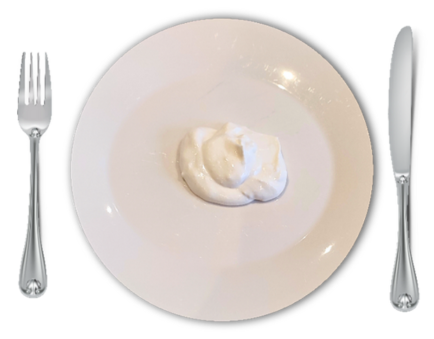


|  | Never or less than once per month | 1-3 per month | Once a week | 2-4 per week | 5-6 per week | Once a day | 2-3 per day | 4-5 per day | 6+ per day | I don’t know what this item is |
| --- | --- | --- | --- | --- | --- | --- | --- | --- | --- | --- |
| ⊗Cream cheeses / soured creams (30g or 2 tablespoons) Examples: Cream cheese Sour cream Crème fraiche Labneh |  |  |  |  |  |  |  |  |  |  |

**Beverages - Water Kefir/Tibicos** **How many times did you consume one serving of water kefir/Tibicos in the past 30 days?**


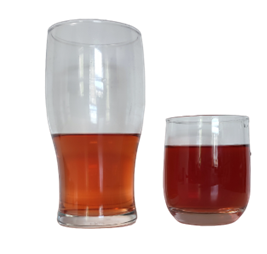


|  | Never or less than once per month | 1-3 per month | Once a week | 2-4 per week | 5-6 per week | Once a day | 2-3 per day | 4-5 per day | 6+ per day | I don’t know what this item is |
| --- | --- | --- | --- | --- | --- | --- | --- | --- | --- | --- |
| ⊗Water Kefir/Tibicos (1 glass or 3/4 of a cup or 175ml or 6 oz) |  |  |  |  |  |  |  |  |  |  |

**Beverages - Kombucha** **How many times did you consume one serving of kombucha in the past 30 days?**


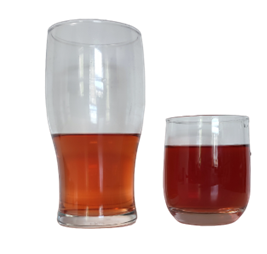


|  | Never or less than once per month | 1-3 per month | Once a week | 2-4 per week | 5-6 per week | Once a day | 2-3 per day | 4-5 per day | 6+ per day | I don’t know what this item is |
| --- | --- | --- | --- | --- | --- | --- | --- | --- | --- | --- |
| ⊗Kombucha (1 glass or 3/4 of a cup or 175ml or 6 oz) |  |  |  |  |  |  |  |  |  |  |

**Beverages - fermented ginger beer** **How many times did you consume one serving of fermented ginger beer (excluding ginger Ale) in the past 30 days?**


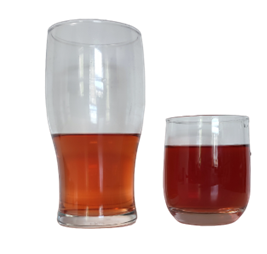


|  | Never or less than once per month | 1-3 per month | Once a week | 2-4 per week | 5-6 per week | Once a day | 2-3 per day | 4-5 per day | 6+ per day | I don’t know what this item is |
| --- | --- | --- | --- | --- | --- | --- | --- | --- | --- | --- |
| ⊗Fermented ginger beer (1 glass or 3/4 of a cup or 175ml or 6 oz) |  |  |  |  |  |  |  |  |  |  |

**Beverages - probiotic sodas** **How many times did you consume one serving of probiotic soda in the past 30 days?**


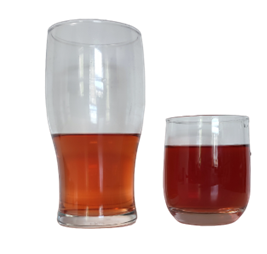


|  | Never or less than once per month | 1-3 per month | Once a week | 2-4 per week | 5-6 per week | Once a day | 2-3 per day | 4-5 per day | 6+ per day | I don’t know what this item is |
| --- | --- | --- | --- | --- | --- | --- | --- | --- | --- | --- |
| ⊗Probiotic soda (1 glass or 3/4 of a cup or 175ml or 6 oz) |  |  |  |  |  |  |  |  |  |  |

**Beverages - probiotic juice shot** **How many times did you consume one serving of probiotic juice shot in the past 30 days?**

|  | Never or less than once per month | 1-3 per month | Once a week | 2-4 per week | 5-6 per week | Once a day | 2-3 per day | 4-5 per day | 6+ per day | I don’t know what this item is |
| --- | --- | --- | --- | --- | --- | --- | --- | --- | --- | --- |
| ⊗Probiotic juice shots (1 bottle) |  |  |  |  |  |  |  |  |  |  |

**Beverages - Beet Kvass** **How many times did you consume one serving of beet kvass in the past 30 days?**


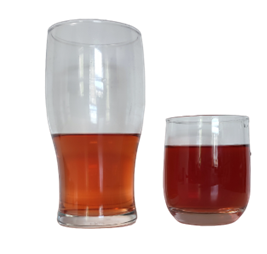


|  | Never or less than once per month | 1-3 per month | Once a week | 2-4 per week | 5-6 per week | Once a day | 2-3 per day | 4-5 per day | 6+ per day | I don’t know what this item is |
| --- | --- | --- | --- | --- | --- | --- | --- | --- | --- | --- |
| ⊗Beet kvass (1 glass or 3/4 of a cup or 175ml or 6 oz) |  |  |  |  |  |  |  |  |  |  |

**Fermented Fish**  Remember to include fermented fish or fermented shellfish when you have them **on their own** and when you use them **as part of recipes**(e.g., pepperoni on pizza). **How many times did you consume one serving of fermented fish or fermented shellfish in the past 30 days?**


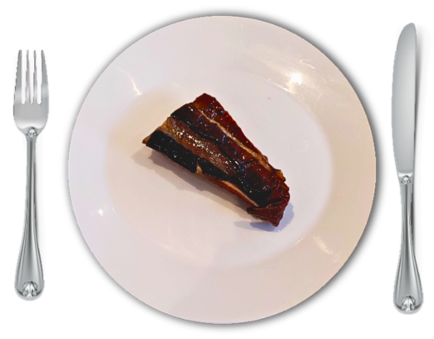


|  | Never or less than once per month | 1-3 per month | Once a week | 2-4 per week | 5-6 per week | Once a day | 2-3 per day | 4-5 per day | 6+ per day | I don’t know what this item is |
| --- | --- | --- | --- | --- | --- | --- | --- | --- | --- | --- |
| Fermented fish or fermented shellfish (56g or 2oz or 1 small tin) Examples: Surstromming, soused herring, fermented shrimp |  |  |  |  |  |  |  |  |  |  |

**Fermented Meats - Salami**   Remember to include salami when you have it **on its own** and when you use it **as part of recipes**(e.g., pepperoni on pizza). **How many times did you consume one serving of Salami in the past 30 days?**


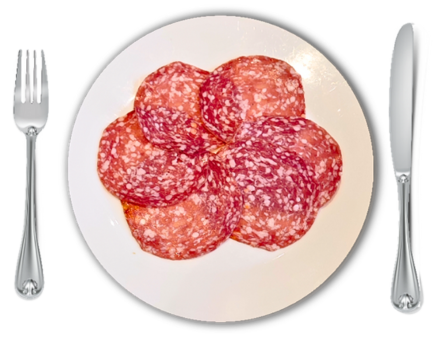


|  | Never or less than once per month | 1-3 per month | Once a week | 2-4 per week | 5-6 per week | Once a day | 2-3 per day | 4-5 per day | 6+ per day | I don’t know what this item is |
| --- | --- | --- | --- | --- | --- | --- | --- | --- | --- | --- |
| ⊗Salami (4 slices or 30g or 1oz) |  |  |  |  |  |  |  |  |  |  |

**Fermented Meats - Prosciutto or *"Prosciutto crudo"***   Remember to include Prosciutto when you have it **on its own** and when you use it **as part of recipes**(e.g., pepperoni on pizza). **How many times did you consume one serving of Prosciutto in the past 30 days?**


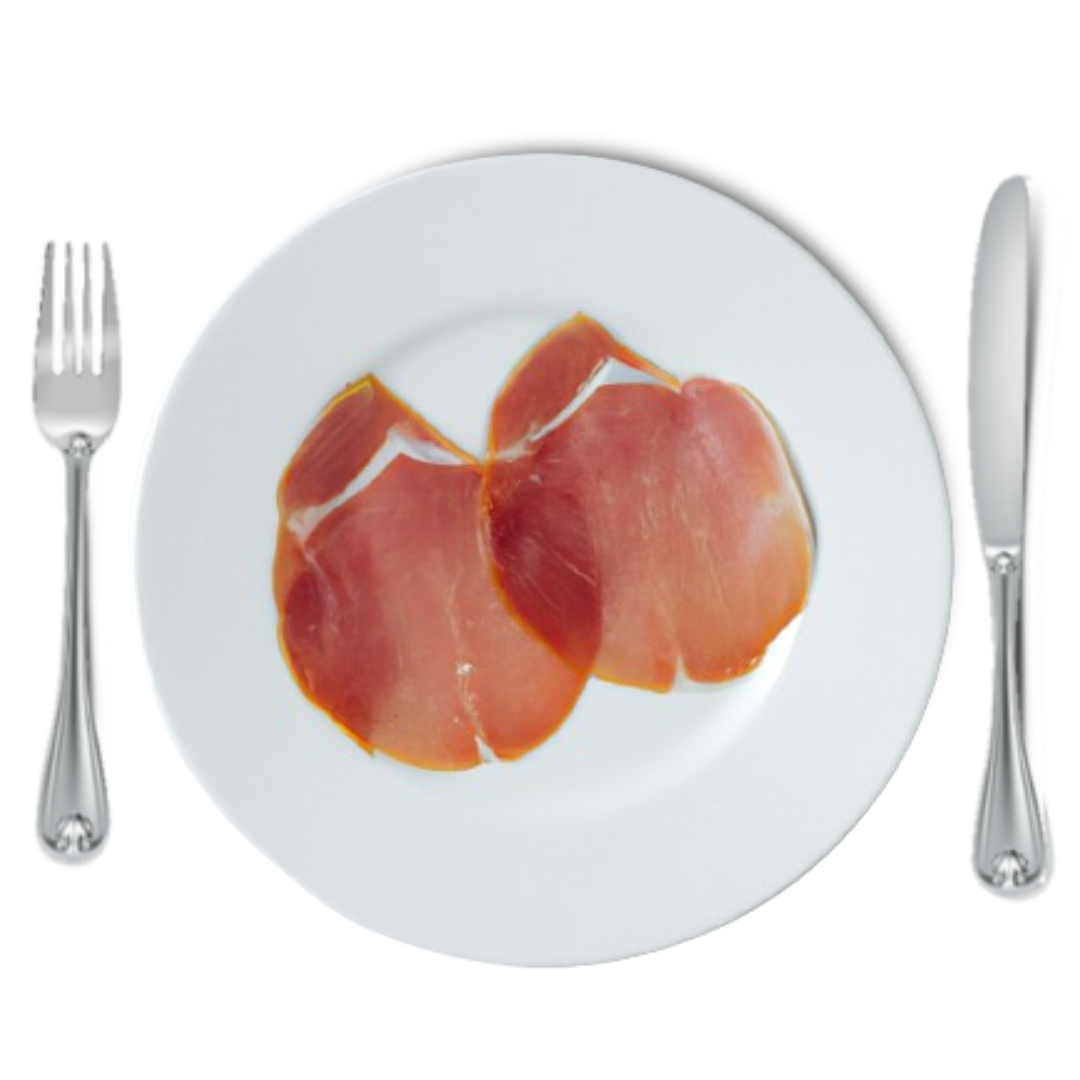


|  | Never or less than once per month | 1-3 per month | Once a week | 2-4 per week | 5-6 per week | Once a day | 2-3 per day | 4-5 per day | 6+ per day | I don’t know what this item is |
| --- | --- | --- | --- | --- | --- | --- | --- | --- | --- | --- |
| ⊗Prosciutto (2 slices or 30g or 1oz) |  |  |  |  |  |  |  |  |  |  |

**Fermented Meats - Chorizo**   Remember to include Chorizo when you have them **it on its own** and when you use it **as part of recipes**(e.g., pepperoni on pizza). **How many times did you consume one serving of Chorizo in the past 30 days?**


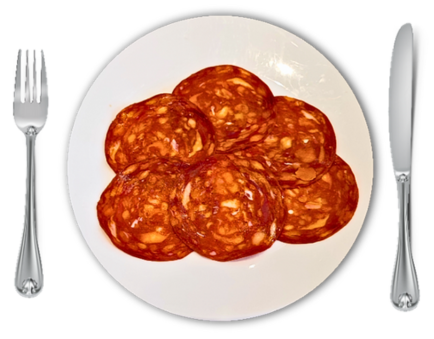


|  | Never or less than once per month | 1-3 per month | Once a week | 2-4 per week | 5-6 per week | Once a day | 2-3 per day | 4-5 per day | 6+ per day | I don’t know what this item is |
| --- | --- | --- | --- | --- | --- | --- | --- | --- | --- | --- |
| ⊗Chorizo (4 slices or 30g or 1oz) |  |  |  |  |  |  |  |  |  |  |

**Fermented Meats - Pepperoni**   Remember to include pepperoni when you have them **on their own** and when you use them **as part of recipes**(e.g., pepperoni on pizza). **How many times did you consume one serving of Pepperoni in the past 30 days?**


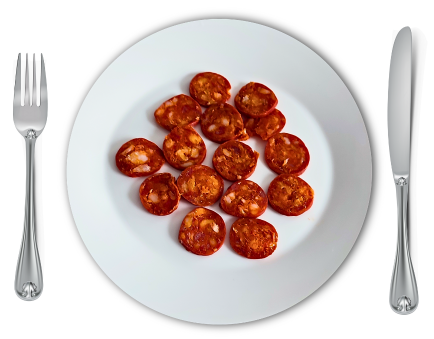


|  | Never or less than once per month | 1-3 per month | Once a week | 2-4 per week | 5-6 per week | Once a day | 2-3 per day | 4-5 per day | 6+ per day | I don’t know what this item is |
| --- | --- | --- | --- | --- | --- | --- | --- | --- | --- | --- |
| ⊗Pepperoni (15 slices or 30g or 1oz) |  |  |  |  |  |  |  |  |  |  |

**Fermented Meats - Summer sausage**   Remember to include summer sausage when you have it **on its own**and when you use it **as part of recipes**(e.g., pepperoni on pizza). **How many times did you consume one serving of summer sasauge in the past 30 days?**


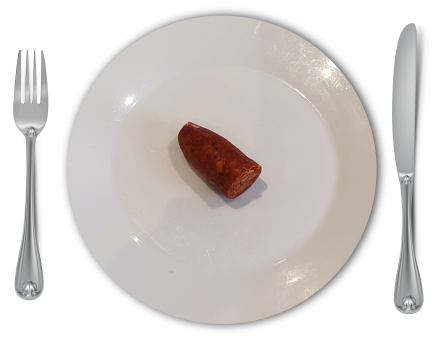


|  | Never or less than once per month | 1-3 per month | Once a week | 2-4 per week | 5-6 per week | Once a day | 2-3 per day | 4-5 per day | 6+ per day | I don’t know what this item is |
| --- | --- | --- | --- | --- | --- | --- | --- | --- | --- | --- |
| ⊗Summer sausage (4 slices or 30g or 1oz) |  |  |  |  |  |  |  |  |  |  |

**Fermented Meats - Lebanon bologna**   Remember to include Lebanon bologna when you have it **on its own** and when you use it **as part of recipes**(e.g., pepperoni on pizza). **How many times did you consume one serving of Lebanon bologna in the past 30 days?**


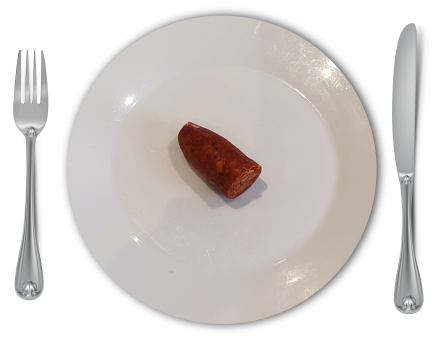


|  | Never or less than once per month | 1-3 per month | Once a week | 2-4 per week | 5-6 per week | Once a day | 2-3 per day | 4-5 per day | 6+ per day | I don’t know what this item is |
| --- | --- | --- | --- | --- | --- | --- | --- | --- | --- | --- |
| ⊗Lebanon bologna (4 slices or 30g or 1oz) |  |  |  |  |  |  |  |  |  |  |

**Fermented soy products - Natto** Remember to include Natto when you have it **on its own** and when you use it **as part of recipes (**e.g., stir fry, Natto on rice) **How many times did you consume one serving of fresh or frozen natto in the past 30 days?**


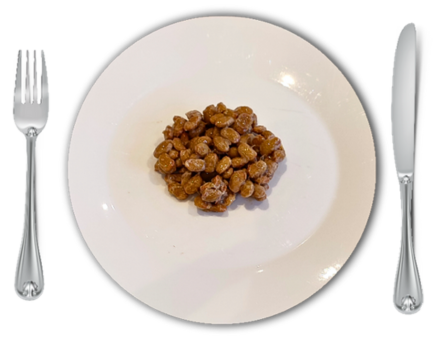


|  | Never or less than once per month | 1-3 per month | Once a week | 2-4 per week | 5-6 per week | Once a day | 2-3 per day | 4-5 per day | 6+ per day | I don’t know what this item is |
| --- | --- | --- | --- | --- | --- | --- | --- | --- | --- | --- |
| Fresh or frozen natto (3 tbsp or about 45g) |  |  |  |  |  |  |  |  |  |  |

**Fermented soy products - Tempeh** Remember to include Tempeh when you have it **on its own** and when you use it **as part of recipes (**e.g., tempeh stir fry) **How many times did you consume one serving of tempeh in the past 30 days?**


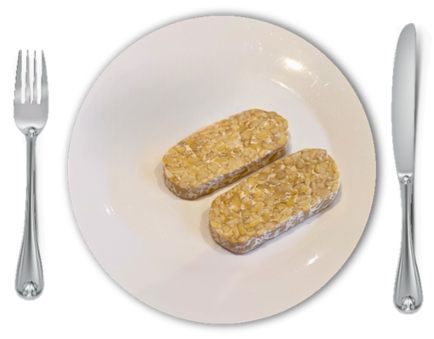


|  | Never or less than once per month | 1-3 per month | Once a week | 2-4 per week | 5-6 per week | Once a day | 2-3 per day | 4-5 per day | 6+ per day | I don’t know what this item is |
| --- | --- | --- | --- | --- | --- | --- | --- | --- | --- | --- |
| Tempeh (3 oz or about 80g) |  |  |  |  |  |  |  |  |  |  |

**Fermented vegetables and Fruits - Kimchi** Remember to include Kimchi when you have it **on its own** and when you use it **as part of recipes** (e.g., chicken and kimchi, olives in a salad etc.) **How many times did you consume one serving of kimchi in the past 30 days?**


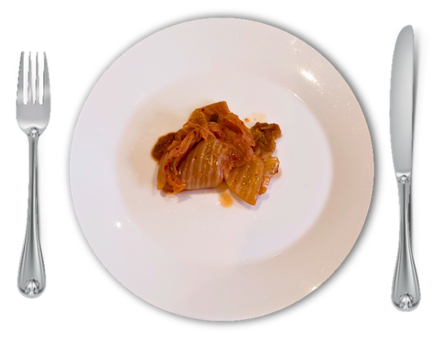


|  | Never or less than once per month | 1-3 per month | Once a week | 2-4 per week | 5-6 per week | Once a day | 2-3 per day | 4-5 per day | 6+ per day | I don’t know what this item is |
| --- | --- | --- | --- | --- | --- | --- | --- | --- | --- | --- |
| Kimchi (40g or 1.5 oz or 1/4 of a cup) |  |  |  |  |  |  |  |  |  |  |

**Fermented vegetables and Fruits - Sauerkraut** Remember to include Sauerkraut when you have it **on its own** and when you use it **as part of recipes** (e.g., chicken and kimchi, olives in a salad etc.) **How many times did you consume one serving of sauerkraut in the past 30 days?**


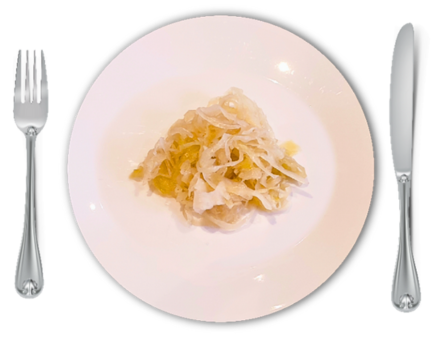


|  | Never or less than once per month | 1-3 per month | Once a week | 2-4 per week | 5-6 per week | Once a day | 2-3 per day | 4-5 per day | 6+ per day | I don’t know what this item is |
| --- | --- | --- | --- | --- | --- | --- | --- | --- | --- | --- |
| ⊗Sauerkraut (40g or 1.5 oz or 1/4 of a cup) |  |  |  |  |  |  |  |  |  |  |

**Fermented vegetables and Fruits - Olives** Remember to include olives when you have them **on their own** and when you use them **as part of recipes** (e.g., chicken and kimchi, olives in a salad etc.). **How many times did you consume one serving of olives in the past 30 days?**


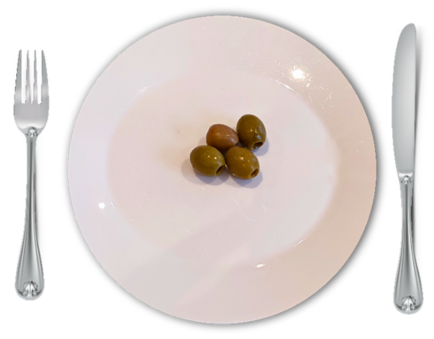


|  | Never or less than once per month | 1-3 per month | Once a week | 2-4 per week | 5-6 per week | Once a day | 2-3 per day | 4-5 per day | 6+ per day | I don’t know what this item is |
| --- | --- | --- | --- | --- | --- | --- | --- | --- | --- | --- |
| Olives/table olives (15g or 0.5 oz or 4 medium olives) |  |  |  |  |  |  |  |  |  |  |
