# Supplementary material for: Development and Validation of a Food Frequency Questionnaire to Assess Fermented Food Consumption in Adults
Source: J Hum Nutr Diet. 2026 Jan 14;39(1):e70183. doi: 10.1111/jhn.70183 (PMC12801178; doi:10.1111/jhn.70183)
Supplement: Supplementary file 2 — Supplementary Figure 1. Percentage contributions of selected fermented food groups to total fermented food consumption reported in the FFIQ by country. Supplementary Figure 2. Fermented vegetables. Supplementary Figure 3. Fermented dairy intake. Supplementary Figure 4. Fermented beverages intake. Supplementary Figure 5. Fermented meats intake. Supplementary Figure 6. Cheese (total) intake. [file JHN-39-0-s001.docx]

**Supplementary figures – Descriptive plots**

**Supplementary Figure 1.** Percentage contributions of selected fermented food groups to total fermented food consumption reported in the FFIQ by country.

**
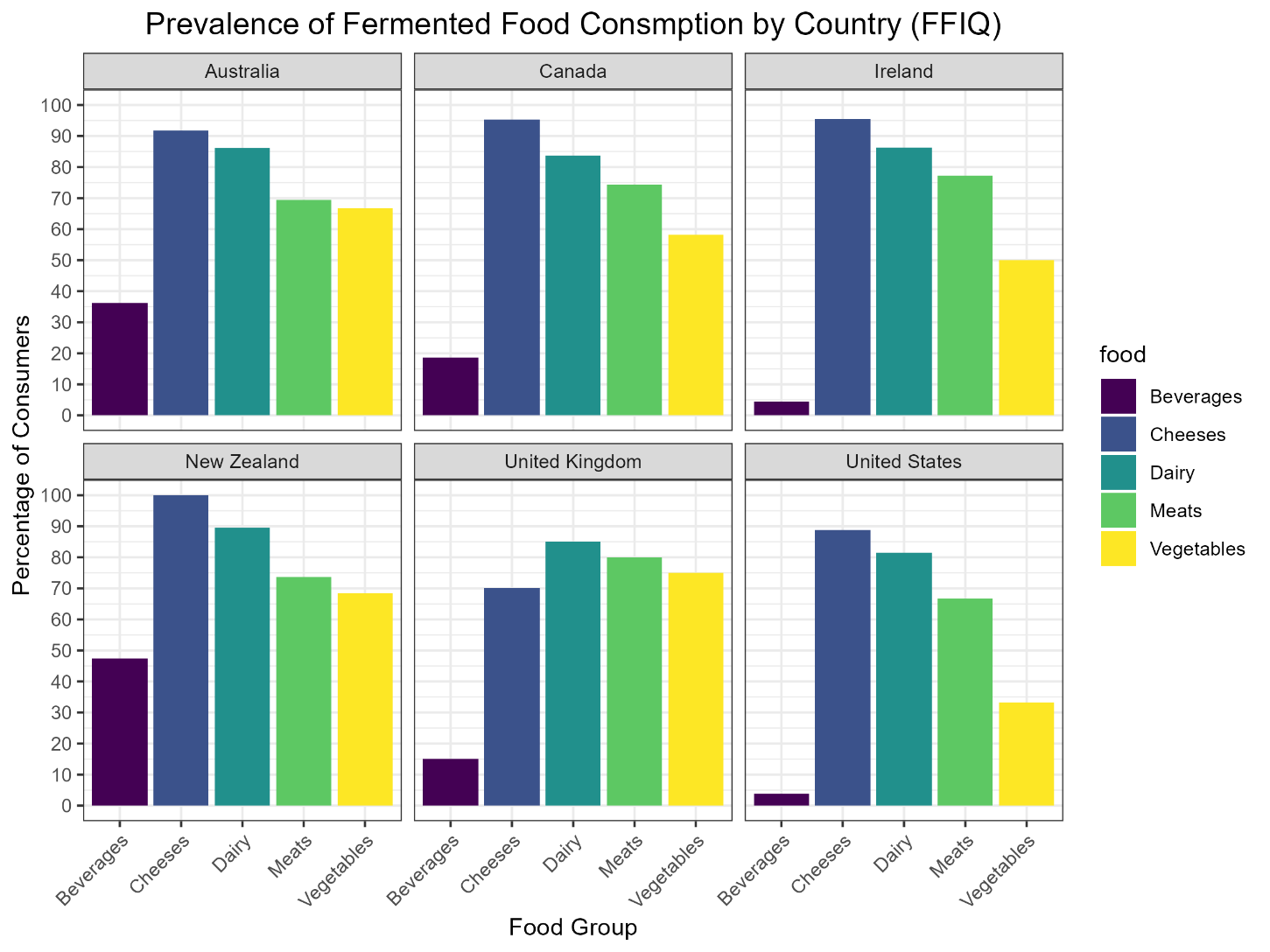
**

**Supplementary figures - Bland Altman plots**

**Supplementary Figure 2.** Fermented vegetables.


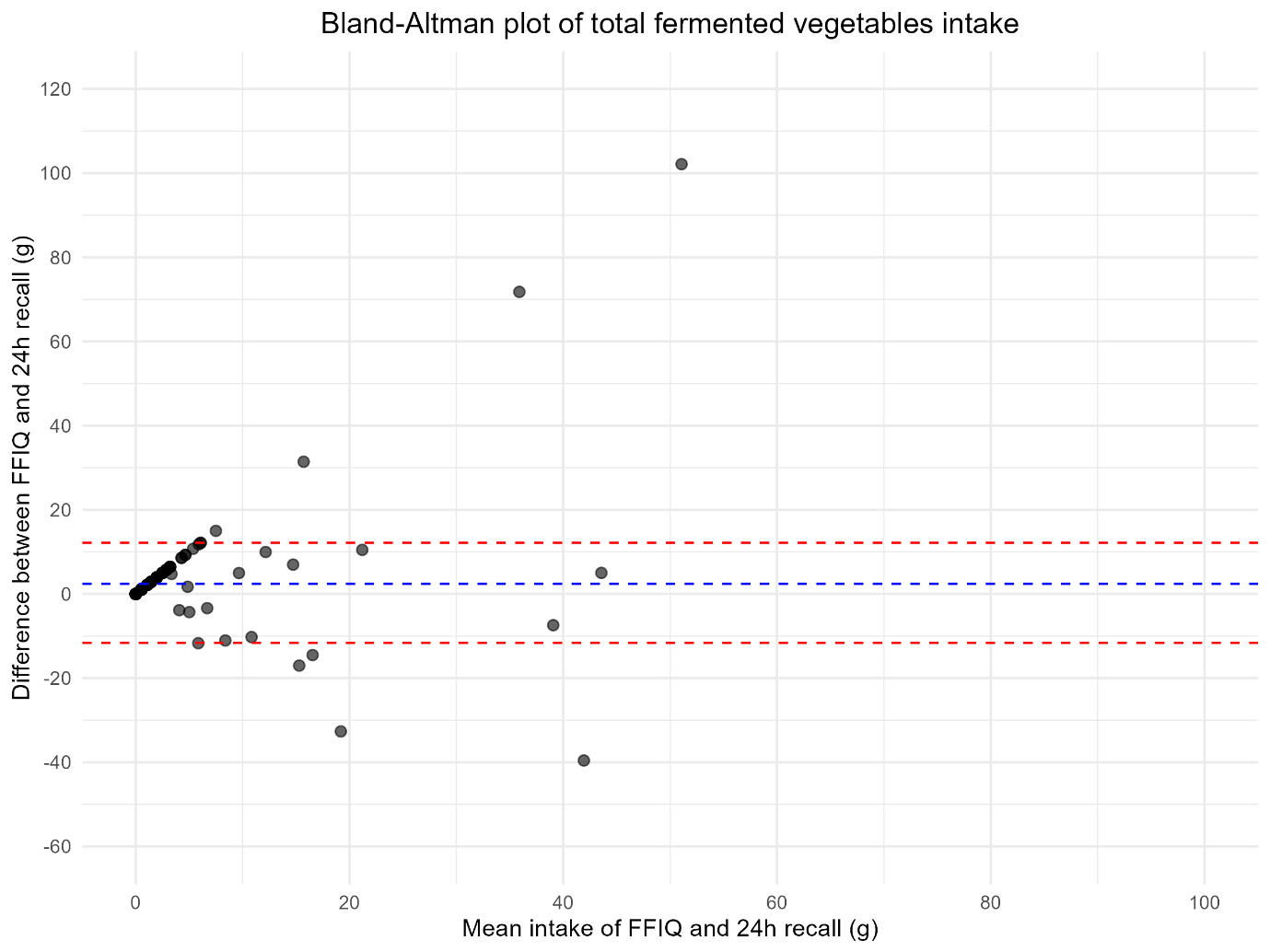


**Supplementary Figure 3.** Fermented dairy intake.


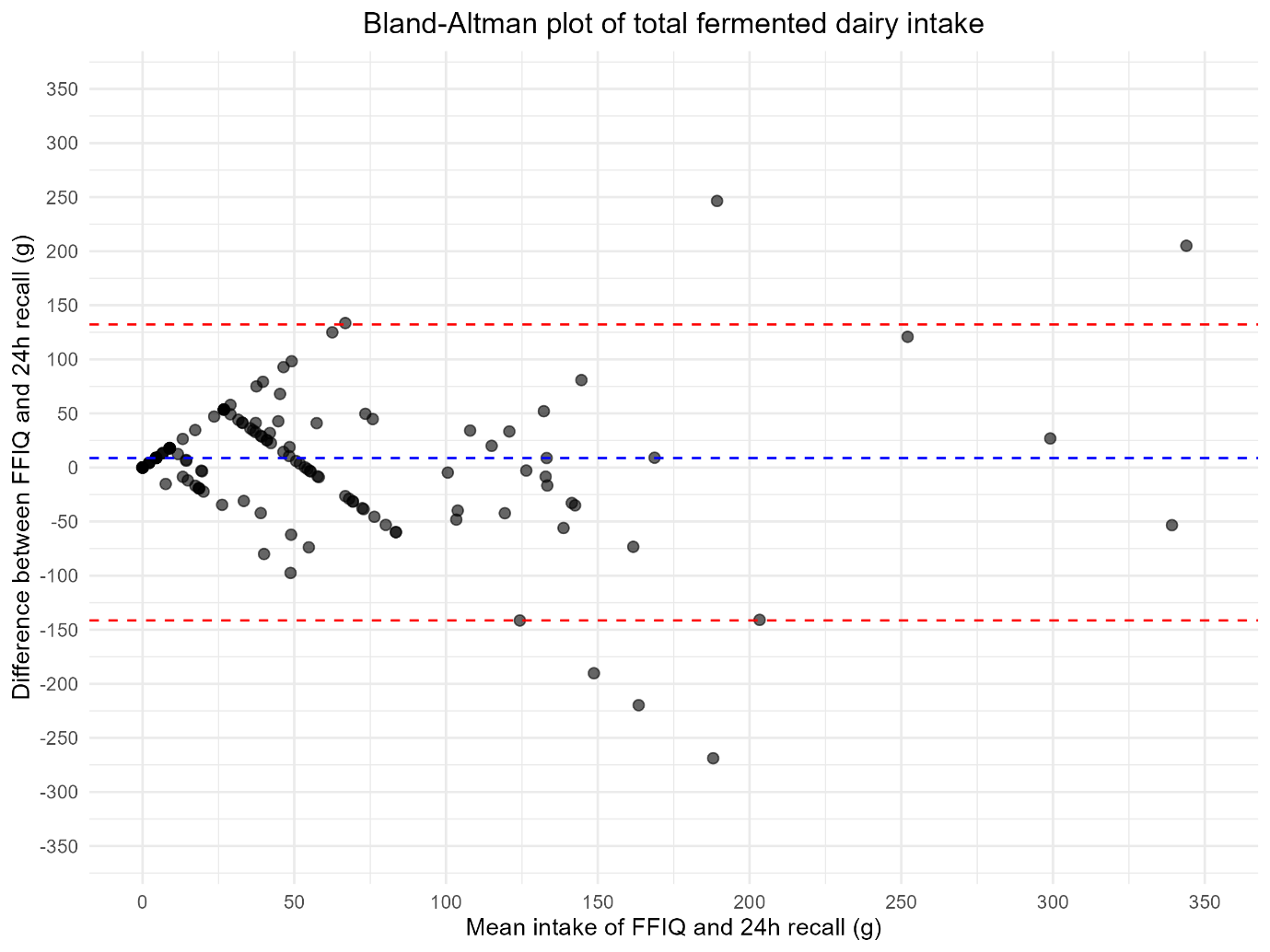


**Supplementary Figure 4.** Fermented beverages intake.


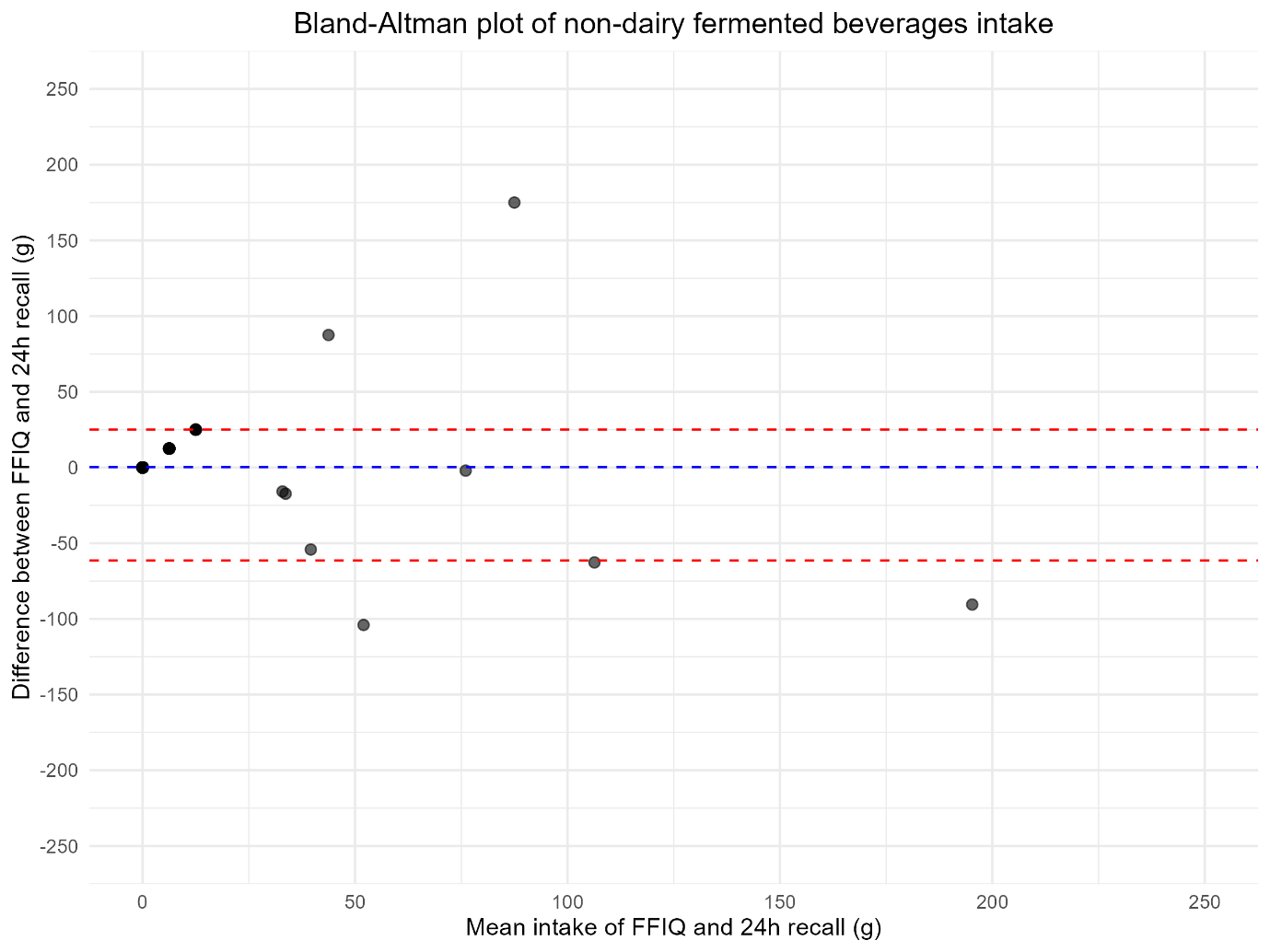


**Supplementary Figure 5.** Fermented meats intake.


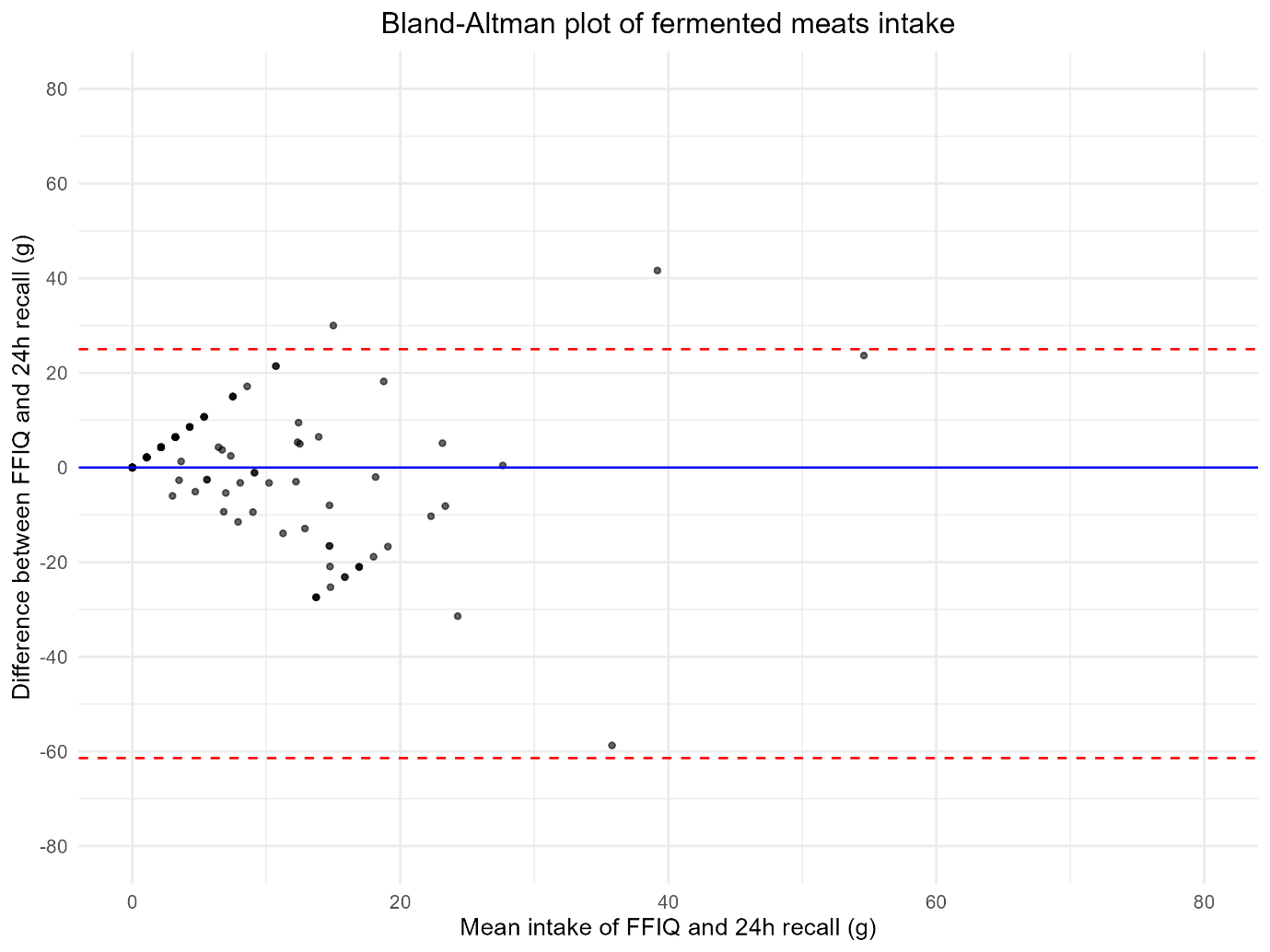


**Supplementary Figure 6.** Cheese (total) intake.


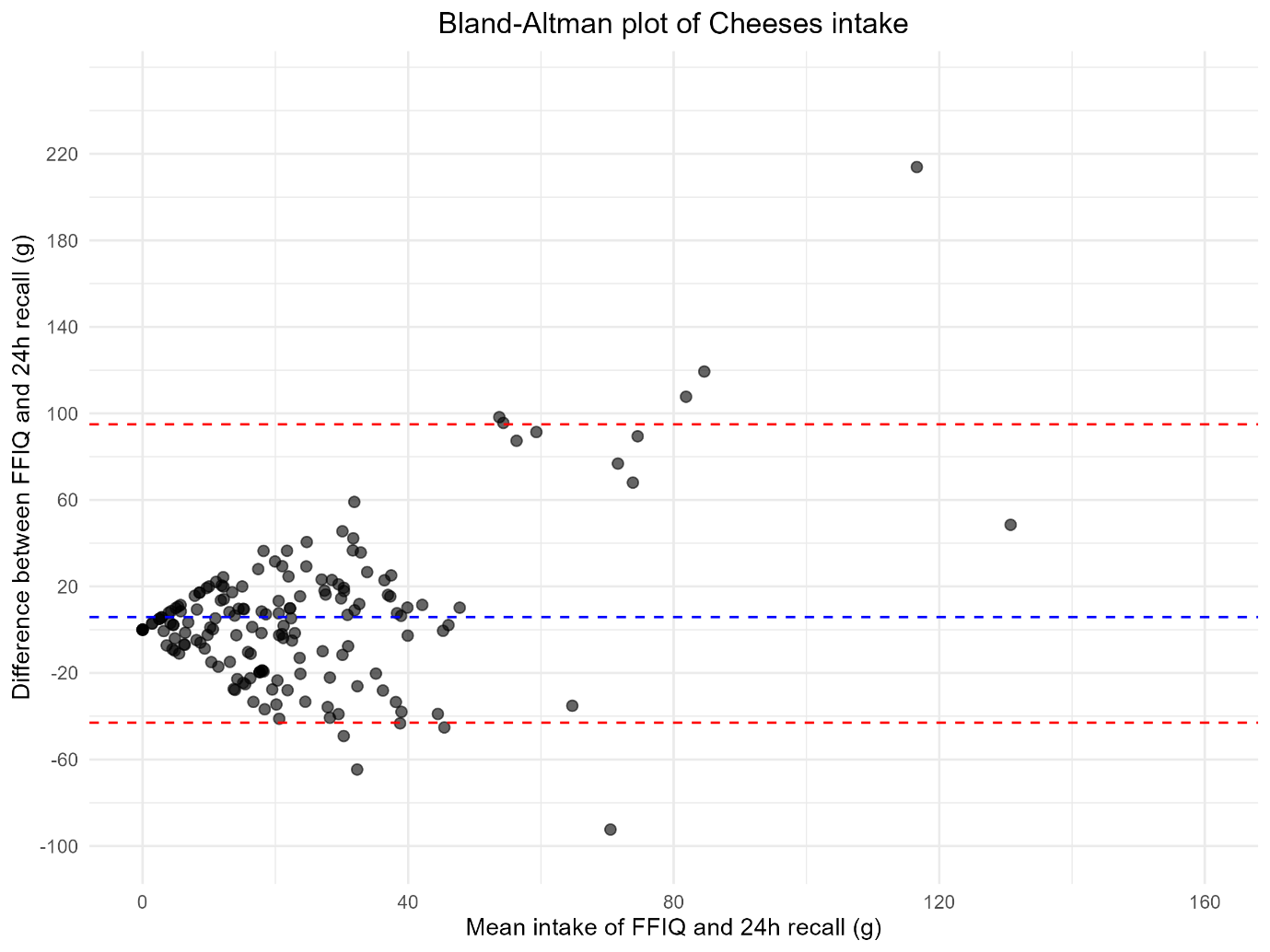


**Supplementary information 1**. SCOFF questionnaire

The SCOFF questionnaire is a brief, validated screening tool for identifying individuals at risk for eating disorders. It comprises five questions measured using a dichotomous yes/no response option. Scores are obtained by summing the number of ‘Yes’ responses. According to the original validation study, the SCOFF provided 100% sensitivity for anorexia and bulimia (95% confidence interval 96.9% to 100%) and a specificity of 87.5% (79.2% to 93.4%)

- Do you make yourself **S**ick (induce vomiting) because you feel uncomfortably full?
- Do you worry you have lost **C**ontrol over how much you eat?
- Have you recently lost more than **O**ne stone (approximately 15 pounds or 6.5 kilograms) in a 3-month period?
- Do you believe yourself to be **F**at when others say you are too thin?
- Would you say that **F**ood dominates your life?

Reference: Morgan JF, Reid F, Lacey JH. The SCOFF questionnaire: assessment of a new screening tool for eating disorders. BMJ 1999;319:1467–8. https://doi.org/10.1136/bmj.319.7223.1467.

| **Supplementary Table 1.** Sensitivity analyses | | | | | |
| --- | --- | --- | --- | --- | --- |
| **Stratification group** | **Variable** | **Spearman's Rho** | **CI lower** | **CI upper** | **P value** |
| **Age** |  |  |  |  |  |
| Lowest tertile | Total intake | 0.51 | 0.30 | 0.68 | <0.001 |
| Middle tertile | Total intake | 0.49 | 0.25 | 0.67 | <0.001 |
| Highest tertile | Total intake | 0.61 | 0.42 | 0.76 | <0.001 |
| **Country** |  |  |  |  |  |
| United States | Total intake | 0.68 | 0.35 | 0.86 | 0.001 |
| Canada | Total intake | 0.57 | 0.33 | 0.75 | <0.001 |
| United Kingdom | Total intake | 0.53 | 0.14 | 0.78 | 0.012 |
| Ireland | Total intake | 0.36 | -0.02 | 0.65 | 0.063 |
| Australia | Total intake | 0.52 | 0.23 | 0.72 | 0.001 |
| New Zealand | Total intake | 0.62 | 0.23 | 0.84 | 0.006 |
| **SCOFF** |  |  |  |  |  |
| Low SCOFF | Total intake | 0.55 | 0.43 | 0.66 | <0.001 |
| **Sex** |  |  |  |  |  |
| M | Total intake | 0.48 | 0.30 | 0.63 | <0.001 |
| F | Total intake | 0.65 | 0.50 | 0.76 | <0.001 |
| Age tertiles, Lowest tertile =19-33y, Middle tertile = 34-42y, Highest tertile = 43-60y; Low SCOFF = total score <2; M = Male, F = Female. | | | | | |

**Supplementary table 2**. Additional demographics.

| **Characteristic** | **N = 167***^1^* |
| --- | --- |
| **Current smoker** | 9 (5.4%) |
| **Physical activity level** |  |
| Light - Walking, exercise 1-3 days a week | 64 (38.3%) |
| Moderate - 3 days/week of hard exercise or 5+ days of light exercise | 68 (40.7%) |
| None or little/no regular exercise | 16 (9.6%) |
| Very active – 5+ days a week of hard exercise | 19 (11.4%) |
| **Usual diet type** |  |
| Regular/no specific diet | 131 (78.4%) |
| Plant-based (includes vegan, vegetarian and flexitarian) | 14 (8.4%) |
| Low calorie | 9 (5.4%) |
| Low carbohydrate | 5 (3%) |
| Other | 8 (4.8%) |
| **Highest education level attained** |  |
| Doctorate degree (PhD/other) | 5 (3.0%) |
| Graduate degree (MA/MSc/MPhil/other) | 28 (17%) |
| High school diploma/A-levels | 23 (14%) |
| No formal qualifications | 1 (0.6%) |
| Secondary education (e.g. GED/GCSE) | 10 (6.0%) |
| Technical/community college | 20 (12%) |
| Undergraduate degree (BA/BSc/other) | 80 (48%) |
| **Current employment status** |  |
| Full-Time | 108 (65%) |
| Not in paid work (e.g., homemaker, retired or disabled) | 16 (9.6%) |
| Other | 4 (2.4%) |
| Part-Time | 31 (19%) |
| Unemployed (and job-seeking) | 8 (4.8%) |
| **Income** |  |
| $1-60,000 | 83 (50%) |
| $60,000-120,000 | 53 (32%) |
| $120,000-180,000 | 17 (10%) |
| $180,000 and above | 12 (7%) |
| No income | 2 (1%) |
| **SCOFF score total** |  |
| 0-1 | 138 (82.6%) |
| ≥ 2 | 29 (17.4%) |
|  |  |
| **BMI** | 26.4 (22.8, 31.4) |
| Missing values | 75 |
| *^1^* n (%); Median (Q1, Q3) | |
